# A mathematical model of the role of aggregation in sonic hedgehog signalling

**DOI:** 10.1371/journal.pcbi.1008562

**Published:** 2021-02-22

**Authors:** Daniel J. A. Derrick, Kathryn Wolton, Richard A. Currie, Marcus John Tindall

**Affiliations:** 1 Department of Mathematics and Statistics, University of Reading, Whiteknights, Reading, United Kingdom; 2 Syngenta, Jealott’s Hill International Research Centre, Bracknell, Berkshire, United Kingdom; 3 Institute of Cardiovascular and Metabolic Research, University of Reading, Whiteknights, Reading, United Kingdom; University of Pittsburgh, UNITED STATES

## Abstract

Effective regulation of the sonic hedgehog (Shh) signalling pathway is essential for normal development in a wide variety of species. Correct Shh signalling requires the formation of Shh aggregates on the surface of producing cells. Shh aggregates subsequently diffuse away and are recognised in receiving cells located elsewhere in the developing embryo. Various mechanisms have been postulated regarding how these aggregates form and what their precise role is in the overall signalling process. To understand the role of these mechanisms in the overall signalling process, we formulate and analyse a mathematical model of Shh aggregation using nonlinear ordinary differential equations. We consider Shh aggregate formation to comprise of multimerisation, association with heparan sulfate proteoglycans (HSPG) and binding with lipoproteins. We show that the size distribution of the Shh aggregates formed on the producing cell surface resembles an exponential distribution, a result in agreement with experimental data. A detailed sensitivity analysis of our model reveals that this exponential distribution is robust to parameter changes, and subsequently, also to variations in the processes by which Shh is recruited by HSPGs and lipoproteins. The work demonstrates the time taken for different sized Shh aggregates to form and the important role this likely plays in Shh diffusion.

## Author summary

The sonic hedgehog (Shh) pathway is vital for normal development in a wide variety of species and its activity is strictly regulated to ensure correct spatiotemporal patterning of numerous developing tissues. Shh signalling requires the formation of Shh aggregates, formed on producing cells via a range of different mechanisms, that then diffuse to receiving cells. We formulate and analyse a mathematical model of the most well described mechanisms, namely monomer multimerisation, and recruitment of Shh by heparan sulfate proteoglycans and lipoproteins. Our results illustrate a distribution of the size and quantities of aggregates formed by these mechanisms. We found that as a consequence of competition between the mechanisms for Shh monomers the shape distribution of Shh aggregates resembles an exponential distribution. We also found the distribution to be robust to both parameter changes and variations to the processes by which mechanisms recruit Shh. We report that our approach and subsequent results demonstrate that these mechanisms act in synergy allowing Shh to aggregate in various quantities with diverse diffusive abilities. We postulate that this regulation contributes significantly to aid precision in signalling for Shh in areas of development.

## Introduction

The Hedgehog (Hh) family of proteins are fundamental to the organisation and direction of tissue patterning in embryonic development in a wide variety of animal species [1–4]. Sonic hedgehog (Shh), one isoform of the Hh family, also functions in adult organisms, for example in the maintenance of stem cells or wound repair [1]. The aberrant activation of Hh pathways is implicated in tumourigenesis; it is estimated that approximately 25% of cancer deaths in humans are tied to Hh signalling [2].

The generation of Shh protein, its subsequent transport and reception at target cells is a strictly regulated spatiotemporal process. Hh proteins undergo autocatalysis and covalent modification with cholesterol and palmitate at the C and N terminals, respectively [3] within producing cells. The modified protein is transported to the cell surface where various aggregation mechanisms are then utilised to form different size aggregates which diffuse to receiving cells containing the Shh receptor Patched-1 (Ptch) and trigger a signalling cascade. Therefore, Shh aggregate formation and diffusion must be tightly regulated to ensure precise spatial distribution of the Shh gradient over a strict developmental time window. Indeed, disruptions to the normal processing of Shh can impede standard dispersal processes and distort gradient formation at critical stages of morphogenesis. This can lead to developmental malformations [4–6].

Early research into Shh revealed its ability to form multimeric aggregates [7]. This indicated that Shh could be mobilised by enclosing the lipid heads of the protein within the multimeric core, negating their inherent hydrophobicity and attachment to the producing cell surface. Formation of these multimeric aggregates has been demonstrated to increase the potency of Shh signalling and aid its local distribution to cells during transport [8].

Both vertebrate and *D*. *melanogaster* Hh have also been shown to bind to lipoproteins. Studies in which lipophorin has been experimentally reduced have shown consequent disruption to Hh signalling [9] and, interestingly, led to the subsequent accumulation of Hh around the producing cell, indicating that Hh diffusion had been critically impaired. Shh has also been demonstrated to have the ability to bind vertebrate lipoproteins [10]. Lipoproteins are proposed to provide a surface in which Shh can effectively bury in and conceal its hydrophobic lipid heads. These are proposed then to diffuse and deliver Shh to a receiving cell. This concept is harmonious with the finding that the Shh receptor Ptch can function as a lipoprotein receptor [11]. There are still however, significant knowledge gaps in the underlying mechanisms of such a process, such as how Shh initially associates with the particles or even how it later disassociates and forms a complex with Ptch. Data suggest that modification of Shh by cholesterol is a mandatory process required for interaction with lipoproteins, which suggests lipid modification may provide one explanation this role for the processed protein [10].

The process by which large multimers are formed on the cell surface has remained unclear, however some data suggest that HSPGs may play a role and function as a large scaffold during their formation [12–13]. Indeed Vyas et al. [14], found that in *D*. *melanogaster*, Hh requires organisation into small multimers before interacting with HSPGs. In addition, disrupting the synthesis of HSPGs in *D*. *melanogaster* will significantly impair the movement of Hh within the developing embryo [15–18]. Shh binds HSPGs via the conserved Cardin-Weintraub motif and disrupting this interaction can impair signalling [12–13,19–21]. A full understanding of the role that HSPGs perform in Shh signalling is yet to be determined. In addition to a possible role in the formation of multimers, HSPGs have been implicated in conjunction with sheddases and the glycoprotein Scube-2 for the release of unlipidated and therefore soluble Shh from the surface of cells [21–23]. Additionally, in *D*. *melanogaster* HSPGs have been found to support the transport of Hh together with lipophorin, the insect equivalent to vertebrate lipoproteins [18].

The collective role that multimerisation and Shh association with lipoproteins and HSPGs play in Shh aggregate formation and subsequent translocation, however, has yet to be fully understood.

The majority of mathematical modelling of Hh proteins has been focused on exploring the dynamics of the morphogen gradient throughout the development of a region, such as the limb bud or neural tube. Modelling of this has mostly relied on simulating Shh diffusion; some have explored the application of an adaptive Shh expression boundary [24] and many integrate Shh receiving cell components [25–28]. Shh pathway models have indicated that some of the temporal behaviour involved in Shh diffusion is the result of adaptation within the pathway, potentially via feedback loops within the pathway [25–30]. Some have incorporated transport and diffusion processes into their models [26–27,30] which were reported to benefit the system by regulating dispersal and potentially contributing to the temporal response for Shh during development. Koleva and colleagues investigated Shh cluster formation on the surface of COS-7 cells and their dispersal into the surrounding extracellular medium [31]. Using confocal microscopy, they recorded Shh cluster volumes against their frequency over 24 hours and showed that the cell associated and dispersed volumes frequency data followed the form of an exponential distribution. Subsequent mathematical modelling assumed cluster formation occurred via multimerisation in a pairwise manner (Smoluchowski coagulation [32]); previously shown to tend to a solution expressed in terms of gamma functions which is exponential in shape [33]. Koleva and colleagues subsequently fitted this function to their data to determine the underlying parameters. We observe that an exponential distribution is a realisation of a Poisson process in which events occur at a continuous and random (independent) rate, which do not have any memory associated with them. Mechanistically it demonstrates decreasing likelihood of an event occurring. In the context of multimer formation as observed by Koleva et al. [31], this relates to increased numbers of smaller sized aggregates, in comparison to larger ones.

**Fig 1:**
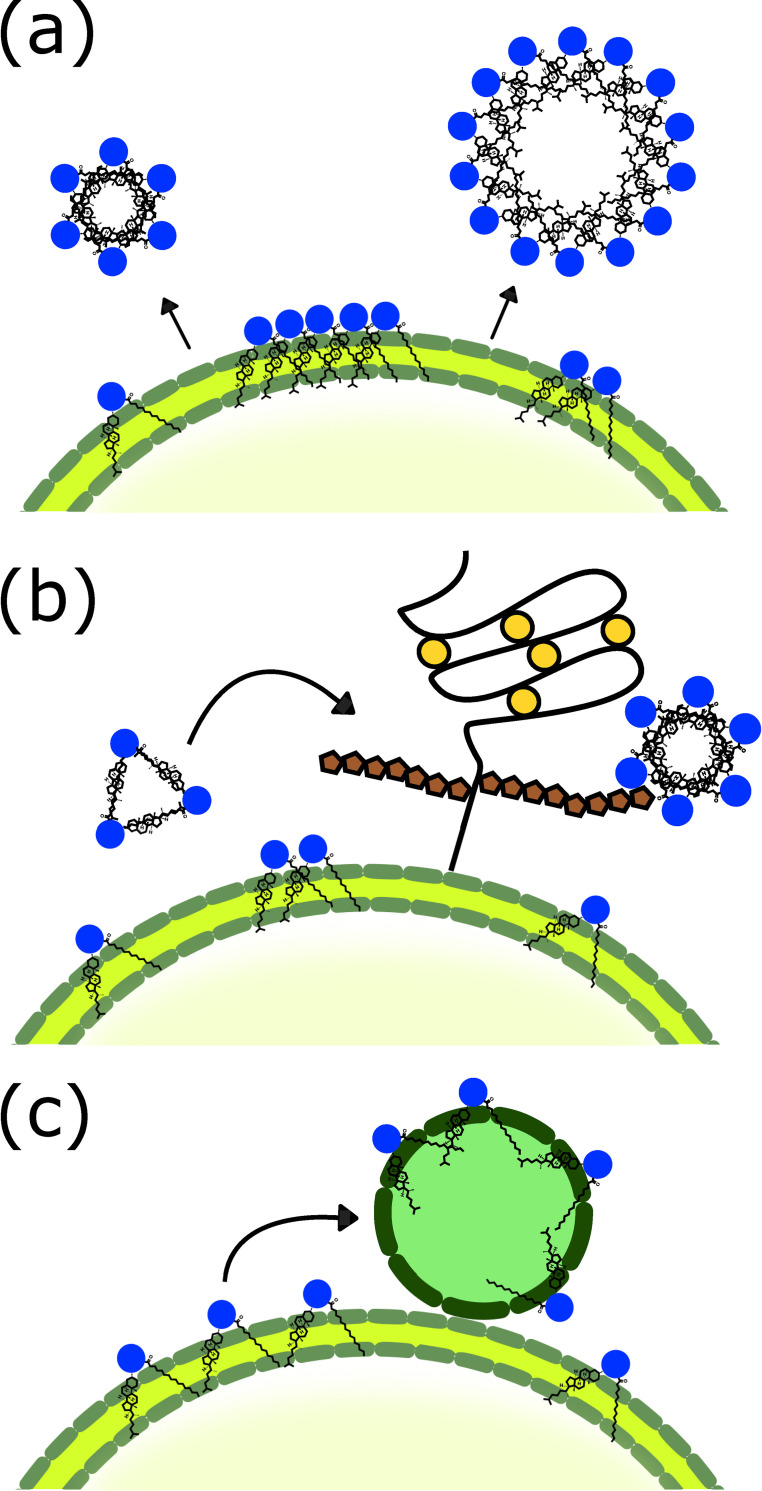
The formation of Shh aggregates. Multimerisation (a) of Shh occurs by protein monomers aggregating together on the producing cell surface. Alternatively, small Shh multimers may be recruited to (b) heparan sulfate proteoglycans (HSPGs) before disassociating from the surface of the cell. In (c) Lipoprotein transportation is facilitated by the binding of lipoproteins to the producing cell surface whereby Shh monomers can individually associate with the particle before it disassociates from the cell surface.

In this work we compare and contrast the roles of multimerisation, HSPG and lipoprotein aggregation via the formulation and analysis of a mathematical model of Shh aggregation. Whilst other mechanisms driving aggregation have been suggested [34–36], including exosomes, we found these mechanisms currently lack the minimum degree of detail that would be necessary for mathematical modelling. The mechanisms of multimerisation, HSPG and lipoprotein aggregate formation are considered to function as depicted in Fig 1. We investigate whether certain processes perform a significantly more prevalent role than others in forming aggregates, and examine the effect competition has on this process.

## Materials and methods

A series of nonlinear ordinary differential equations (ODEs) were formulated describing each of the Shh aggregation processes (multimerisation, HSPGs and lipoproteins) via the law of mass action. Full details can be found in S1 Text. Key processes underlying each transport mechanism were identified from the literature and were used to inform our model as follows.

Aggregate formation via each process occurs on the surface of a single producing cell as shown in Fig 1. We observe that [37] reported the largest Shh aggregate with a molecular weight of 4000 kDa in filtration studies, corresponding approximately to 200 Shh in a complex, whilst [31] calculated that 90% of their Shh monomer population were associated with multimers consisting of 32 monomers or less. Whilst simulations were run for aggregates comprising up to 200 monomers, the vast majority of aggregates (97%) consisted of 40 monomers or less (see S2 Table, S3 Table, S4 Fig, S5 Fig and S5 Text). As such, results here are plotted for aggregates consisting of up to 40 monomers or less, and the respective percentages associated with aggregates comprising more than 40 monomers are reported in Tables 1 and 2. We consider simulations for 24 hours and at steady-state (approximately 35–40 hours).

**Table 1:** Percentage breakdown of mechanisms that form cell associated Shh aggregates shown in Fig 2. The percentage of each mechanism responsible for forming the respective size aggregate in terms of the total number of Shh protein monomers and aggregates formed.

	1	2	3	4	5	6	7	8	9	10	
**Monomers**	20.02	-	-	-	-	-	-	-	-	-	
**Multimers**	-	6.11	2.00	1.29	0.64	0.42	0.26	0.19	0.12	0.09	
**HSPGs**	0.00	3.73	1.26	2.32	1.45	1.72	1.37	1.40	1.23	1.19	
**Lipoproteins**	5.29	4.87	4.47	4.10	3.72	3.33	2.91	2.48	2.04	1.61	
	11	12	13	14	15	16	17	18	19	20	
**Monomers**	-	-	-	-	-	-	-	-	-	-	
**Multimers**	0.10	0.10	0.10	0.09	0.09	0.08	0.08	0.07	0.07	0.07	
**HSPGs**	1.03	0.99	0.87	0.82	0.73	0.68	0.61	0.56	0.50	0.45	
**Lipoproteins**	1.22	0.88	0.61	0.40	0.25	0.15	0.09	0.05	0.02	0.01	
	21	22	23	24	25	26	27	28	29	30	
**Monomers**	-	-	-	-	-	-	-	-	-	-	
**Multimers**	0.06	0.06	0.06	0.06	0.05	0.05	0.05	0.05	0.05	0.04	
**HSPGs**	0.41	0.37	0.33	0.29	0.26	0.23	0.20	0.18	0.16	0.14	
**Lipoproteins**	0.01	0.00	0.00	0.00	0.00	0.00	0.00	0.00	0.00	0.00	
	31	32	33	34	35	36	37	38	39	40	>40
**Monomers**	-	-	-	-	-	-	-	-	-	-	
**Multimers**	0.04	0.04	0.04	0.04	0.04	0.04	0.04	0.04	0.04	0.04	2.26
**HSPGs**	0.12	0.11	0.09	0.08	0.07	0.06	0.05	0.04	0.04	0.03	0.16
**Lipoproteins**	0.00	0.00	0.00	0.00	0.00	0.00	0.00	0.00	0.00	0.00	0.00

**Table 2:** Percentage breakdown of mechanisms that form dispersed Shh aggregates as depicted in Fig 3. The percentage of each mechanism responsible for forming the respective size aggregate in terms of the total number of Shh protein monomers and aggregates formed.

	1	2	3	4	5	6	7	8	9	10	
**Monomers**	29.02	-	-	-	-	-	-	-	-	-	
**Multimers**	-	9.74	3.54	2.54	1.42	1.04	0.71	0.58	0.42	0.35	
**HSPGs**	0.00	2.85	1.04	1.70	1.12	1.25	1.02	1.02	0.90	0.86	
**Lipoproteins**	5.58	4.69	3.88	3.16	0.52	1.97	1.50	1.10	0.79	0.54	
	11	12	13	14	15	16	17	18	19	20	
**Monomers**	-	-	-	-	-	-	-	-	-	-	
**Multimers**	0.34	0.32	0.28	0.26	0.24	0.22	0.20	0.19	0.17	0.16	
**HSPGs**	0.70	0.67	0.57	0.53	0.46	0.42	0.37	0.33	0.29	0.25	
**Lipoproteins**	0.36	0.23	0.14	0.08	0.05	0.03	0.01	0.01	0.00	0.00	
	21	22	23	24	25	26	27	28	29	30	
**Monomers**	-	-	-	-	-	-	-	-	-	-	
**Multimers**	0.15	0.14	0.13	0.12	0.12	0.11	0.10	0.10	0.09	0.09	
**HSPGs**	0.22	0.19	0.17	0.15	0.13	0.11	0.10	0.08	0.07	0.06	
**Lipoproteins**	0.01	0.00	0.00	0.00	0.00	0.00	0.00	0.00	0.00	0.00	
	31	32	33	34	35	36	37	38	39	40	>40
**Monomers**	-	-	-	-	-	-	-	-	-	-	
**Multimers**	0.09	0.08	0.08	0.07	0.07	0.07	0.06	0.06	0.06	0.06	1.79
**HSPGs**	0.05	0.04	0.04	0.03	0.03	0.02	0.02	0.02	0.01	0.01	0.05
**Lipoproteins**	0.00	0.00	0.00	0.00	0.00	0.00	0.00	0.00	0.00	0.00	0.00

These are time periods in which Shh signalling in some species will be ongoing, but also periods in which some critical targets will have been specified [38]. It is reported that maximal pathway response will be achieved within this period following initial Shh exposure in the vertebrate neural tube [39–40]. Reviewing the distribution profile at 24 hours also allows us to compare our distributions with that of Koleva and colleagues [31]. Multimer formation is assumed to occur in a pairwise manner given the decreased likelihood of more than two proteins (individually or of larger aggregate sizes) simultaneously being able to aggregate together in the same spatial location. For instance, two monomers may aggregate to form a dimer which itself can then form a larger aggregate, either with a monomer or larger sized multimer. We assume that HSPGs can recruit multimers that consist of up to 10 monomers. This assumption is based upon, in part, the calculation by Whalen et al. who estimated that eight Shh would associate with a single heparin chain [13]. Lipoproteins on the other hand are assumed to recruit monomers individually. This is because Shh is proposed to bind lipoproteins by burying its lipid modifications into the phospholipid layer, which would negate the hydrophobicity of Shh. This means that initial events that solubilise the protein, such as multimerisation, would not be necessary. We further assume lipoprotein formed aggregates do not interact with those formed via multimerisation or HSPGs.

Parameter values were informed as follows. An estimate on the production of monomers by cells was previously given by [24], who utilised a value of 800 Shh per cell per minute in a limb bud model. We used this value to inform a constant expression of Shh for a single cell over a 24-hour period. Given a lack of further experimental data to inform the other model parameter values (reaction rate constants and production rates) we chose to inform our model as follows. Assuming the reaction rate constants of aggregate formation for each mechanism was the same, irrespective of their size, we began by determining the rates of HSPG and lipoprotein production by ensuring that neither mechanism would completely dominate aggregate production. The lower bound for HSPG and lipoprotein particle sources in each case was informed by ensuring the proportion of the affected mechanism (e.g. aggregates formed via multimerisation) contributed less than 10% to the overall number of aggregates at 24 hours. The upper bound in each case was informed when the aggregates formed by the respective mechanism dominated the overall distributions (greater than 90%) at the same time. These values were then adjusted using a sensitivity analysis and fit-by-eye to the Koleva et al. [31] data whilst ensuring all three mechanisms were present as reported in the experimental literature [7,10,12]. This led to the source of HSPGs to monomers being 25:1, which we feel is appropriate. Larger sources of HSPGs were found to be more disruptive to multimerisation due to a greater rate of recruitment. The expression of lipoproteins to monomers was found to be 19.2:1, which we also feel is appropriate. This value was found to allow the lipoproteins to contribute to a reasonable degree of competition for monomers with the multimerisation mechanism, but does not cause lipoproteins to excessively dominate aggregate production. S2 Text and S1 Table details the parameter set obtained.

The model was solved numerically in MATLAB using the stiff ODE solver ode15s [41]. Dynamic frequency distribution plots were produced that depicted the generation of aggregate sizes in time via each of the discussed mechanisms. We also examined the accumulation of aggregates modelled as `dispersed’ aggregates. This distribution at steady-state and after 24 hours has similar resemblance to an exponential distribution.

## Results

**Fig 2:**
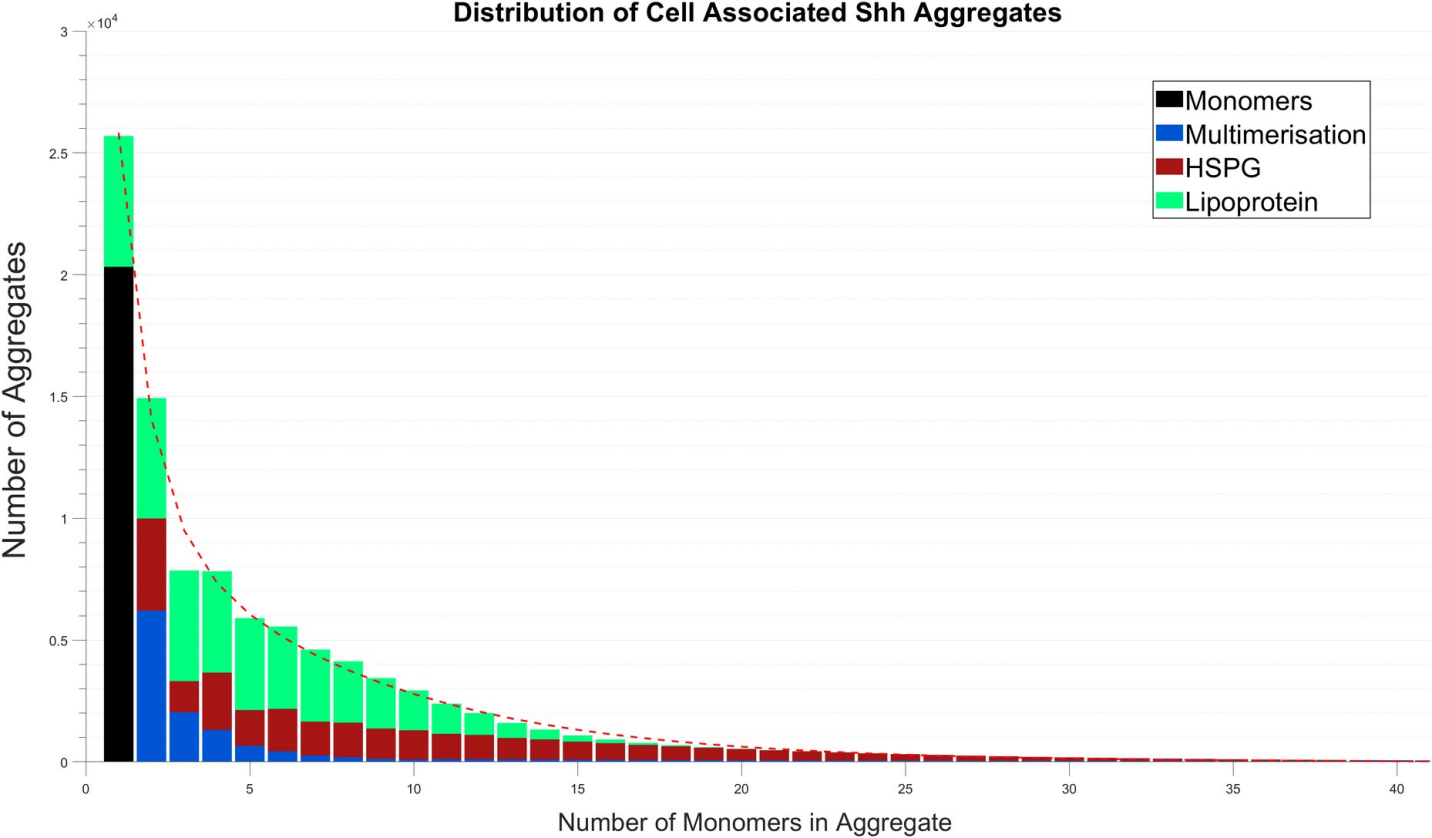
Cell associated Shh distribution: Shh aggregate formation as a result of multimerisation, HSPG and lipoprotein association. Simulation shown is at 24 hours with a breakdown of the total aggregate formation given in Table 1.

**Fig 3:**
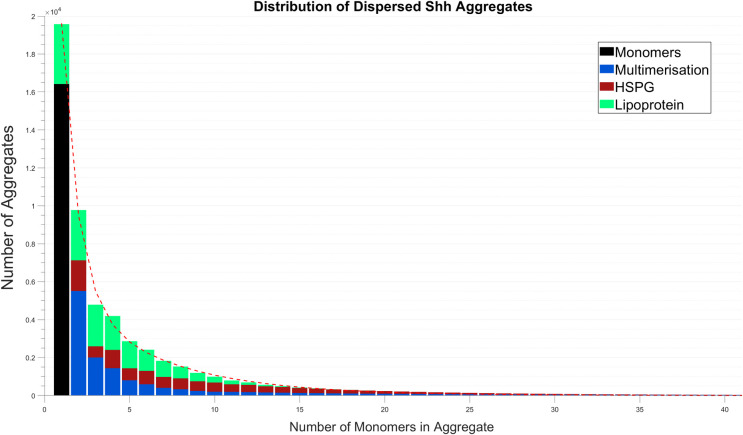
Dispersed Shh distribution: Shh aggregate formation as a result of multimerisation, HSPG and lipoprotein association. Simulation shown is at 24 hours with a breakdown of the total aggregate formation given in Table 2.

Figs 2 and 3 show the contribution that each of the three mechanisms make to Shh aggregation and dispersal at 24 hours. The data shown in Figs 2 and 3 are displayed alternatively in Tables 1 and 2 respectively where we consider the breakdown for each mechanism forming each respective size of aggregate as a percentage of the total number of aggregates and monomers. We observe that cell associated aggregates up to size 20 are generally formed via all three mechanisms (Fig 2). For larger size aggregates of size N (20<N<40), multimerisation and HSPGs dominate. At 24 hours and in steady-state (~36 hours; results detailed in S4 Text) the cell associated distribution exhibits an exponential function shape, there being no distinct difference between the distributions at each of these time points. We note that the results shown in Fig 3 reveal a larger proportion of monomers and small aggregates to have dispersed compared to those that remain cell associated after 24 hours (distribution shown in Fig 2). This is explained by the inability for the dispersed aggregates to interact and further increase in size. Furthermore, the quantity of dispersed smaller aggregates accumulated significantly, prior to the cell associated distribution achieving steady-state.

**Fig 4:**
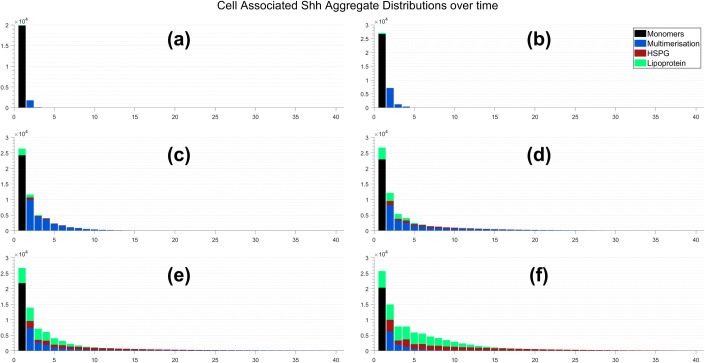
Cell associated Shh aggregate distributions after: (a) 30 minutes; (b) 1 hour; (c) 3 hours; (d) 6 hours; (e) 12 hours; and (f) 24 hours.

**Fig 5:**
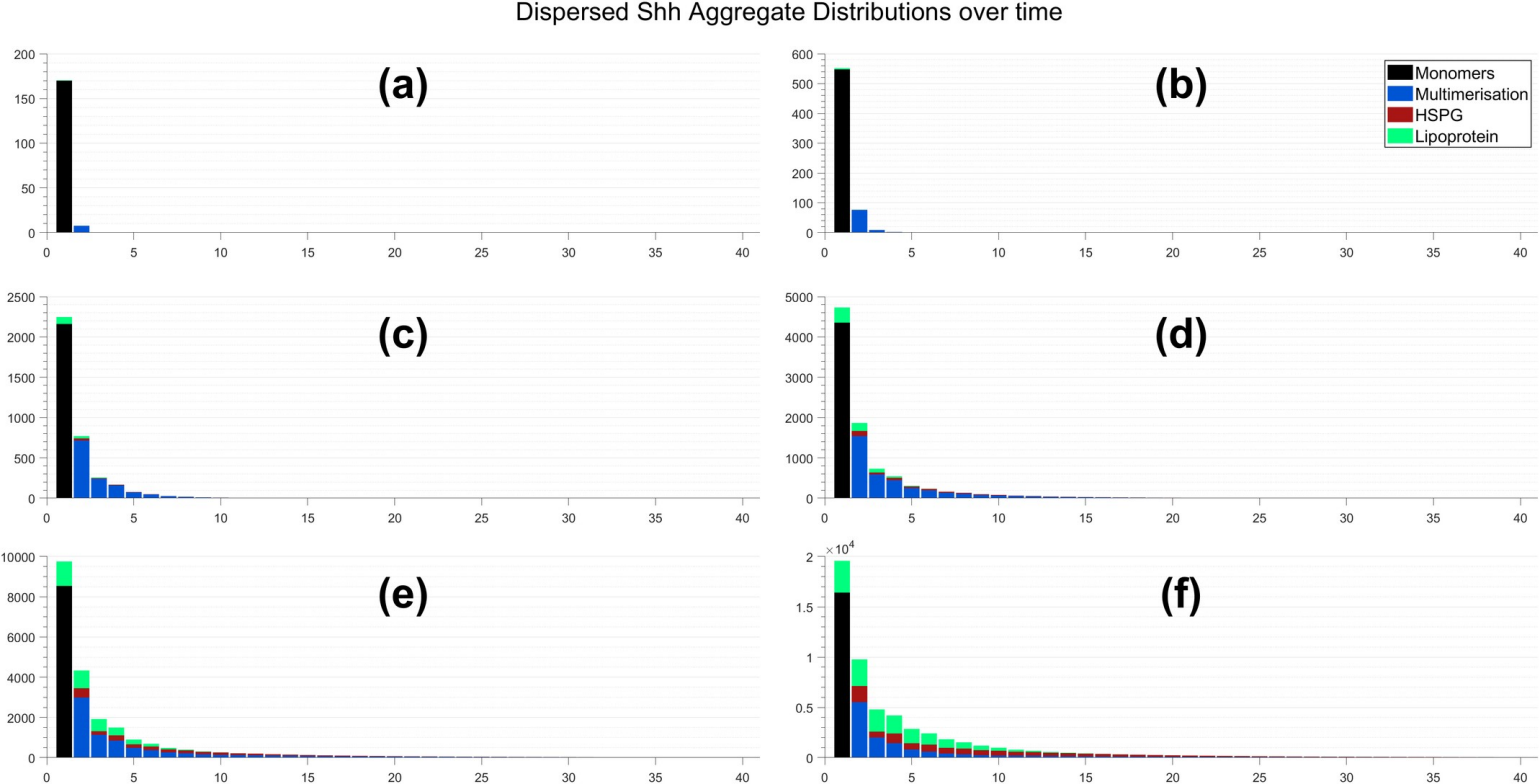
Dispersed Shh aggregate distributions after: (a) 30 minutes; (b) 1 hour; (c) 3 hours; (d) 6 hours; (e) 12 hours; and (f) 24 hours.

The formation of each distribution (cell associated and dispersed) at specific time points demonstrating key changes is shown in Figs 4 and 5, respectively. The overall number of monomers and competition for them plays an important role in affecting how each mechanism forms different size aggregates. This is because each mechanism recruits Shh monomers and multimers in a different way. Multimers dominate the early stages of aggregate formation given that pairwise interactions occur at a considerably more rapid rate than the single monomer recruitment by lipoproteins. In addition, the quantity of lipoprotein and HSPG aggregates at the cell surface is minimal when compared to that of monomers throughout the initial period of aggregate formation. As time progresses, their presence at the surface increases and as such their competition for monomers and multimers is increased; multimers can no longer dominate the formation of smaller size aggregates. However, their pairwise behaviour enables larger aggregates to form quite rapidly, such that their accumulation in intermediate sizes is minimal. We note here that this occurs for an aggregate consisting of 40 Shh proteins given this is the maximal size allowed in our system, as shown in Fig 4(F) and Fig 5(F). Lipoproteins recruit monomers individually and so their aggregate size numbers increase in a step-like manner. This means the increase in lipoprotein aggregate sizes is gradual present as a mechanism in the formation of each size aggregate. A similar result holds for HSPGs; their recruitment of multimers allows them to form a broader array of aggregate sizes in an efficient manner.

We investigated the behaviour of our models when simulated without the accompaniment of the other mechanisms (S3 Text and S1 Fig). When applying the same parameters when all mechanisms were present, we found that the exponential distribution was maintained in the case when only multimerisation was present, a result which agrees with that of Koleva et al. [31]. Given HSPGs depend upon multimerisation we also found that the exponential distribution provides a fair fit to this case. However, in the case of lipoproteins, this is not the case and the exponential distribution is lost. We noted that when lacking the competition of the other processes the mechanisms are capable of forming a significantly broader array of sizes, mostly in minimal quantity. Aggregates in each distribution accumulate at large sizes and are able to do so quite rapidly, given a lack of competition for other mechanisms. These distributions occur when considering the HSPG and lipoprotein distributions individually because production of aggregates is influenced by a now larger effective binding rate and a shift in the ratio of free HSPGs or lipoproteins to available Shh particles. We noted that whilst the distribution for lipoproteins could be made to resemble that of an exponential type distribution, this would be dependent on a large variation in the model parameters. Whilst some mechanisms independently do maintain an exponential distribution, we felt such results were inconsistent with the known experimental observations of multiple mechanisms being responsible for Shh aggregate formation.

### Aggregate distribution stability

We found the general form of an exponential distribution at 24 hours and steady-state was independent of variations in the aggregation mechanisms and their associated parameters.

**Fig 6:**
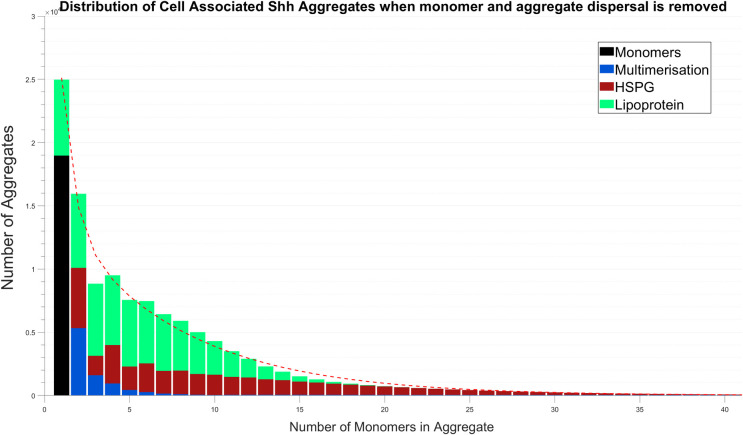
The shape of the Shh aggregate distribution is independent of the rate of dispersal. Here monomer and aggregate dispersal has been removed. Model simulation is at 24 hours.

For instance, reducing or removing the rate of dispersal had a limited effect on the distribution as shown in Fig 6. Differences in the number of aggregates formed by each mechanism, in comparison to the result of Fig 2, are a result of the increased amount of Shh monomers available at the cell surface which would normally disperse. For example, multimer retention led to an increase in the quantity of larger size multimers. This includes the accumulation of the largest aggregate size able to form in our simulations, which signifies a significant increase in binding rates for the mechanism. The increase in multimers and HSPGs available on the cell surface in turn leads to more aggregates being formed via the HSPG mechanism, and also leads to a greater distribution of aggregate sizes by their monomer incorporation. The way in which lipoproteins recruit Shh meant an increase in the number of lipoproteins promoted aggregate formation only moderately, as demonstrated by a minor increase in monomer composition when compared to those of Fig 2. This is a result of the ratio of lipoproteins to Shh monomers meaning the competition for monomers increased. We observed that an increase in the rate of dispersal generally favoured multimerisation. Whilst the quantities and sizes of all aggregates was greatly reduced, the greatest impact was upon HSPG and lipoprotein formed aggregates. The lesser effect on multimerisation was likely due to that mechanism not requiring particle-aggregate interactions.

Increasing and decreasing the source of lipoproteins and HSPGs was found to alter the dominance of the respective mechanism in forming the number of different size aggregates (S7 Fig). Generally, however, the exponential distribution shape was maintained. Increasing the HSPG source term led to an increase in the number of HSPG aggregates, but had the adverse impact of decreasing their impact on the range of aggregate sizes they contributed to. This is because the increased number of HSPG particles increased the rate at which multimers are recruited by the mechanism, and thus reduced the availability of multimers at the cell surface. This however, then reduces the rate of multimer recruitment for the individual HSPGs and therefore in turn prevents further size increases. Interestingly, increasing the source of HSPGs into the system also led to an increase in lipoproteins with greater amounts of Shh associated, which is a result of decreased multimerisation and subsequent competition for Shh monomers. Somewhat unsurprisingly, when the lipoprotein source was increased the multimerisation mechanism was considerably inhibited. This is because multimerisation experiences increased competition for monomers from lipoproteins. Consequently, the efficacy of HSPG production was also adversely affected.

Varying reaction rate parameters for each mechanism did not lead to any unexpected outcomes (S8 Fig). For instance, increasing the reaction rate constant for multimerisation increased the speed by which Shh multimer aggregates were formed. This also led to more multimers being available earlier which could be recruited onto HSPGs. Shh lipoprotein recruitment was inhibited due to a decrease in Shh monomers via increased competition with the multimerisation mechanism. Increasing the reaction rate constants affecting HSPG or lipoprotein Shh binding promoted faster Shh recruitment and the number of larger aggregates formed via these mechanisms. Similar, contrasting outcomes occurred when rate parameters were decreased as detailed in S7 Text.

### Variation in Shh recruitment

**Fig 7:**
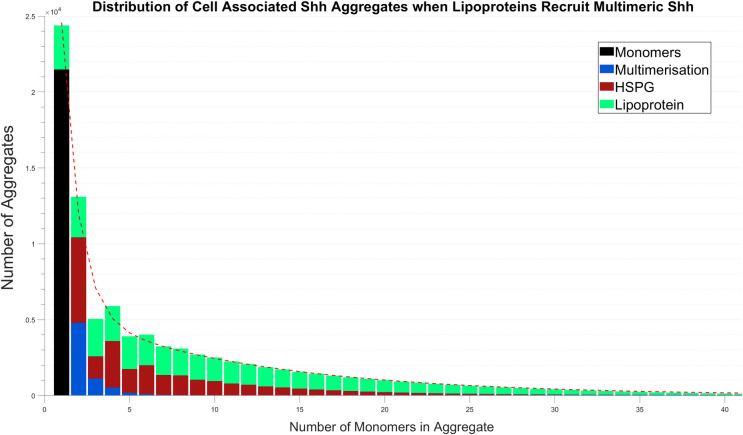
Shh aggregate formation when lipoproteins recruit multimeric Shh. Here lipoproteins recruit multimers consisting of up to five monomers. No significant differences in the observed distribution and the underlying competition between mechanisms was observed in comparison with the results of Fig 2. Model simulation is at 24 hours.

Given the importance of how monomer recruitment effects the contribution of each mechanism to the size of aggregates formed, we sought to consider the effect of relaxing some of the Shh recruitment assumptions for HSPGs and lipoproteins. For instance, we considered whether a variation in the number of Shh proteins recruited onto lipoproteins (as multimers) per interaction could affect our results. We thus considered the effect of lipoproteins being able to recruit multimers consisting of up to five monomers. As shown in Fig 7, the distribution shape remains relatively consistent, with the exception of an increase in the array of lipoprotein aggregate sizes. Interestingly, the profile of lipoprotein aggregates has retained its characteristic array of monotonically decreasing quantities.

**Fig 8:**
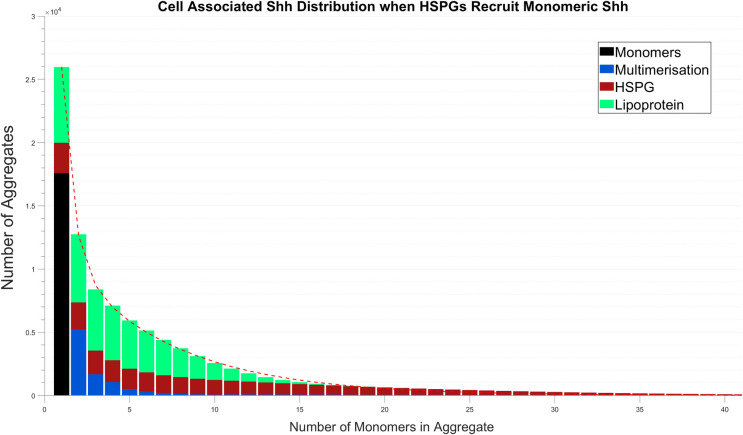
Shh aggregate formation when HSPGs recruit monomeric Shh. Model simulation is at 24 hours.

We also considered the impact of HSPGs being able to recruit monomers in addition to multimers. We originally excluded this ability in our model to reflect the results of Vyas et al. [14] who found that disabling the ability for *D*. *melanogaster* Hh to form multimers would also abrogate its association with HSPGs. Instead of dominating the aggregation process given the high monomer concentration, this change led to the resultant (cell associated) distribution shown in Fig 8 which shows only minor differences when compared to the original distribution (Fig 2). The main differences, albeit minor, include a small increase in the range of HSPG formed aggregate sizes and a reduction in the contribution multimers make to aggregate formation. The lipoprotein mechanism in contrast appears mostly unaffected. This suggests that its ability to recruit monomers was not impacted by the addition of HSPG monomer recruitment. The limited effect this variation has on the final distribution is the result of HSPGs only recruiting multimers consisting of up to 10 monomers and the effect that HSPG monomer recruitment has on multimer formation; a decrease in the number of smaller sized aggregates formed via multimerisation in turn effects HSPG recruitment of multimers.

### Monomer dispersal

In respect of monomer dispersal we observe the following. Whilst some have noted the dispersal of monomer fractions experimentally [7,10,42], it is argued that insoluble processed Shh monomers are not able to overcome membrane binding provided by the lipid modifications [43] and a high amount of energy is required for detachment [44–46]. A possible explanation is that dispersed monomers may be unlipidated. It has been previously suggested that unlipidated Shh may be released from cells and have a synergistic role in supporting signalling [10]. However, it is not yet clear whether cells are able to produce Shh lacking modifications. Experiments have shown that unlipidated Shh has behaviours distinct from its insoluble counterpart and is able for instance to diffuse from the cell in the absence of secretion mechanisms [47]. It is also possible that dispersed soluble Shh monomers are the result of shedding processes [48]. As such, whilst our model includes a description of monomer dispersal, we are also able to consider the effect when this is removed.

The distribution produced following this change (see S6 Fig and S6 Text) has negligible differences in aggregate production when compared to the original distribution (Fig 2). We reason that this is because monomers that disperse do so gradually over a 24-hour period and are present overall in a relatively low amount. For instance, Goetz et al. [49] observed the expression of multimers and monomers in a 9:1 ratio. We find that our results express aggregates at a rate of 2.5:1 over a 24-hour period and in the final hour calculate the ratio to be ~4.2:1. Whilst somewhat less, our results demonstrate that the model produces results within range of those in the literature. We also found that modifying model parameters in attempt to attain a ratio of 9:1 led to a significant disruption to the shape of the distribution and an increase the dominance of certain mechanisms, leaving the remaining ones to contribute minimally to aggregate formation.

Whilst our results suggest that the dispersal of monomeric Shh has an inconsequential effect on aggregate production, the potential importance of Shh monomer dispersal in the context of Shh signalling cannot be discounted. Numerous literature findings have demonstrated that soluble Shh monomers have a higher rate of diffusion than the multimers, and subsequently have an extended range of dispersal. These results may indicate that a pivotal role is executed by Shh monomers during certain stages of development.

## Discussion

We have formulated and analysed a nonlinear ODE model of Shh aggregation for a producing cell. Our system compared the contributions of multimerisation, HSPG and lipoprotein recruitment to the formation of Shh aggregates on the surface of a producing cell and their subsequent dispersal. Analysis of the model showed that in each case the distribution at 24 hours and steady-state would take the form of an exponential distribution. This finding is supported by previous data generated on the volumes of Shh clusters [31], which also resembled this shape. In addition, exponential shaped functions (comprising gamma distributions) have been demonstrated as being solutions to the Smoluchowski coagulation equations [32–33]; these equations describe a generalised system for homogeneous particle aggregation and strongly resemble our model of multimerisation. We have shown that an exponential distribution is maintained in our model when additional Shh aggregation mechanisms are included.

A detailed analysis of the mathematical model revealed that the exponential distribution shape was robust to parameter changes and, furthermore, that this is most likely an outcome of the interconnected behaviour between the mechanisms. Our results suggest that, predominantly, a competition effect occurring between the mechanisms for the recruitment of monomers, and subsequent multimers, drives the appearance and composition of the resultant distribution. A mechanism could be made more dominant in its contribution to aggregate production following the variation of certain parameters, which would often have a corresponding impact, beneficial or inhibitory, on the remaining mechanisms. For instance, the HSPG mechanism would benefit from an increased production of multimers with which it could associate, whereas the multimerisation mechanism could be impeded by an increased rate of Shh recruitment by lipoproteins. Interestingly, in this analysis we determined that the distribution shape was not largely influenced by a varied rate of dispersal, and subsequently, that it was also not significantly altered when dispersal was completely omitted. Instead, we found that the shape was generally maintained but would cause an accumulation of some aggregate populations, such as a considerable abundance of large multimers. This indicated to us that the overall form of the distribution was not determined by aggregate dispersal.

Over time we found that each mechanism varied in their contribution to aggregate production and the size of Shh aggregates formed. Multimerisation was promoted initially as binding rates more directly derive from monomer and multimer availability. This contrasts with both the HSPG and lipoprotein mechanisms as these require the introduction of `free’ HSPG and lipoprotein particles before they can begin to form aggregates. The accumulation of HSPG and lipoprotein aggregates however, as an outcome of their constant introduction over time, enhances the mechanisms by providing a greater and more competitive rate of recruitment. This is a pivotal factor that likely drives larger size aggregate formation throughout later stages of the simulation period.

Further stability of the Shh aggregate exponential distribution shape was demonstrated as we explored variations to recruitment processes. Relaxing the assumption that lipoproteins recruit single Shh monomers, such that they could recruit multimeric Shh allowed the mechanism to produce larger aggregates whilst the remaining mechanisms experienced reduced activity. Generally, however, the promoted function of the lipoprotein mechanism was insignificant and had limited impact on the shape of the overall aggregate distribution. Similarly, following a variation in which HSPG recruitment was altered to be given the ability to recruit monomeric Shh, only minor differences were revealed in the resultant distribution. We predict that this outcome was the result of the stability by which Shh is recruited, given the underlying importance of competition for monomers.

We also investigated the ability for the mechanisms to produce exponential distributions when operating independently. Utilising parameter values derived when all mechanisms were present showed that when each mechanism was considered independently, those of multimerisation and HSPGs independently led to the exponential distribution being maintained. The former result agrees with that of Koleva and colleagues [31], whilst HSPGs are supported by a small distribution of multimers allowing for an exponential shape in the case of the latter. However, for lipoproteins, this distribution was lost, but could be reproduced, with significant changes to parameter values. We question whether given multiple mechanisms have been shown experimentally to be responsible for Shh transport, a single mechanism model is sufficient to reflect the current understanding of Shh aggregation [50].

The different processes implicated in Shh transport may be a significant factor that contributes to and drives the variation in Shh diffusivity. As detailed in S8 Text, S9 Fig, S10 Fig, S11 Fig and S12 Fig, we considered how rates of diffusion may vary for aggregates produced by each mechanism and their sizes as a result of Shh composition. Our calculations suggest that, due to their size, lipoproteins would have a mostly unaffected rate of diffusion as Shh association increases. Conversely, multimers could have a variable diffusive ability as a result of their Shh enrichment.

It is worth noting that the size of the aggregates could increase their rate of uptake by the receiving cell. Literature findings have shown that the range of Shh diffusion is regulated by the expression of Ptch and its co-receptors on the receiving cell surface; in experiments where these receptors are mutant or inactive, Shh has an extended range of diffusion [51–52]. In addition, it has also been shown that Shh lacking lipid modifications, likely to deviate from the standard mechanisms of aggregation [5,7,10,53], can promote the range of diffusion [6,47,54]. It is therefore possible that the process of aggregate production via different mechanisms is important in ensuring Shh transport is strictly maintained. We postulate that aggregates are produced in a coordinated manner, where structures have certain diffusive capabilities within regulated quantities, to enhance specificity of tissue transcription. Therefore, when considering the implications of our results, large aggregate structures with low diffusivity would introduce extensive Shh activity to areas local to the source of production, and the lesser quantities of smaller aggregates with greater diffusivity would induce minor activation at areas more distant. Given the size distributions for the different aggregate forming mechanisms we postulate that: (i) multimerisation is responsible for mostly long range diffusion, given the mechanism dominates the formation of physically smaller aggregates; (ii) HSPG formed aggregates diffuse short, medium and long range distances given they form the full array of aggregate sizes; and (iii) lipoproteins are responsible for short to medium range diffusing aggregates, given their larger sizes. This may also suggest that the variation in size of aggregates produced, and subsequently diffusing, throughout the initial simulation period could have a role in activating transcription at specific distances early in patterning.

Currently a considerable proportion of existing mathematical modelling literature on the Shh pathway is dedicated to understanding the adaptive properties of the morphogenic gradient during development. Our results suggest that aggregate formation could contribute a considerable array of signalling variation to this process and the inclusion of a description of aggregation in future work may aid further modelling of dynamic gradient behaviour. Our work has also indicated a need for more detailed experimental understanding of Shh aggregation at the scale at which aggregates are formed. Furthermore, whilst other mechanisms driving aggregation have been suggested [34–36] we found these mechanisms currently lack the minimum degree of detail that would be necessary for modelling. Thus, future experimental work at more refined scales would allow for not only more detailed data at the resolution of aggregation to be collected, but the relative importance of other mechanisms to be considered.

## Supporting information

S1 Fig Shh aggregate formation as a result of independent mechanisms. (TIFF)

S2 Fig Cell associated Shh distribution at steady-state: Shh aggregate formation as a result of multimerisation, HSPG and lipoprotein association. (TIF)

S3 Fig Dispersed Shh distribution at steady-state: Shh aggregate formation as a result of multimerisation, HSPG and lipoprotein association. (TIF)

S4 Fig Full axis view of the cell associated Shh distribution. (TIF)

S5 Fig Full axis view of the dispersed Shh distribution. (TIF)

S6 Fig Cell associated Shh aggregate formation following the removal of monomer dispersal. (TIF)

S7 Fig Shh aggregate distributions with: (a) half the rate of multimerisation; (b) doubled rate of multimerisation; (c) half the rate of HSPG binding; (d) doubled rate of HSPG binding; (e) half the rate of lipoprotein binding; and (f) double the rate of lipoprotein binding.
(TIF)

S8 Fig Shh aggregate distributions with: (a) half the rate of multimerisation; (b) doubled rate of multimerisation; (c) half the rate of HSPG binding; (d) doubled rate of HSPG binding; (e) half the rate of lipoprotein binding; and (f) double the rate of lipoprotein binding.
(TIF)

S9 Fig Diagram of Shh multimers. (TIFF)

S10 Fig Diagram of Shh aggregation by association with lipoproteins. (EPS)

S11 Fig Diagram of Shh aggregation by association with HSPGs. (EPS)

S12 Fig Approximated diffusion coefficients for the aggregates produced by multimerisation, HSPG and lipoprotein recruitment. (TIF)

S1 Text Model derivation. (PDF)

S2 Text Estimating parameter values. (PDF)

S3 Text Individual mechanism distributions. (PDF)

S4 Text Steady-state distribution. (PDF)

S5 Text Full view of distribution figure axis. (PDF)

S6 Text Monomer dispersal removed. (PDF)

S7 Text Sensitivity analysis. (PDF)

S8 Text Diffusion coefficient approximation. (PDF)

S1 Table Parameters for the aggregation model. (PDF)

S2 Table Percentage breakdown of mechanisms that form the steady- state cell associated Shh aggregate distribution as shown in S2 Fig. (PDF)

S3 Table Percentage breakdown of mechanisms that form the steady- state dispersed Shh aggregate distribution as shown in S3 Fig. (PDF)
