# Supplementary figures and images for: A mathematical model of the role of aggregation in sonic hedgehog signalling
Source: PLoS Comput Biol. 2021 Feb 22;17(2):e1008562. doi: 10.1371/journal.pcbi.1008562 (PMC7932509; doi:10.1371/journal.pcbi.1008562)

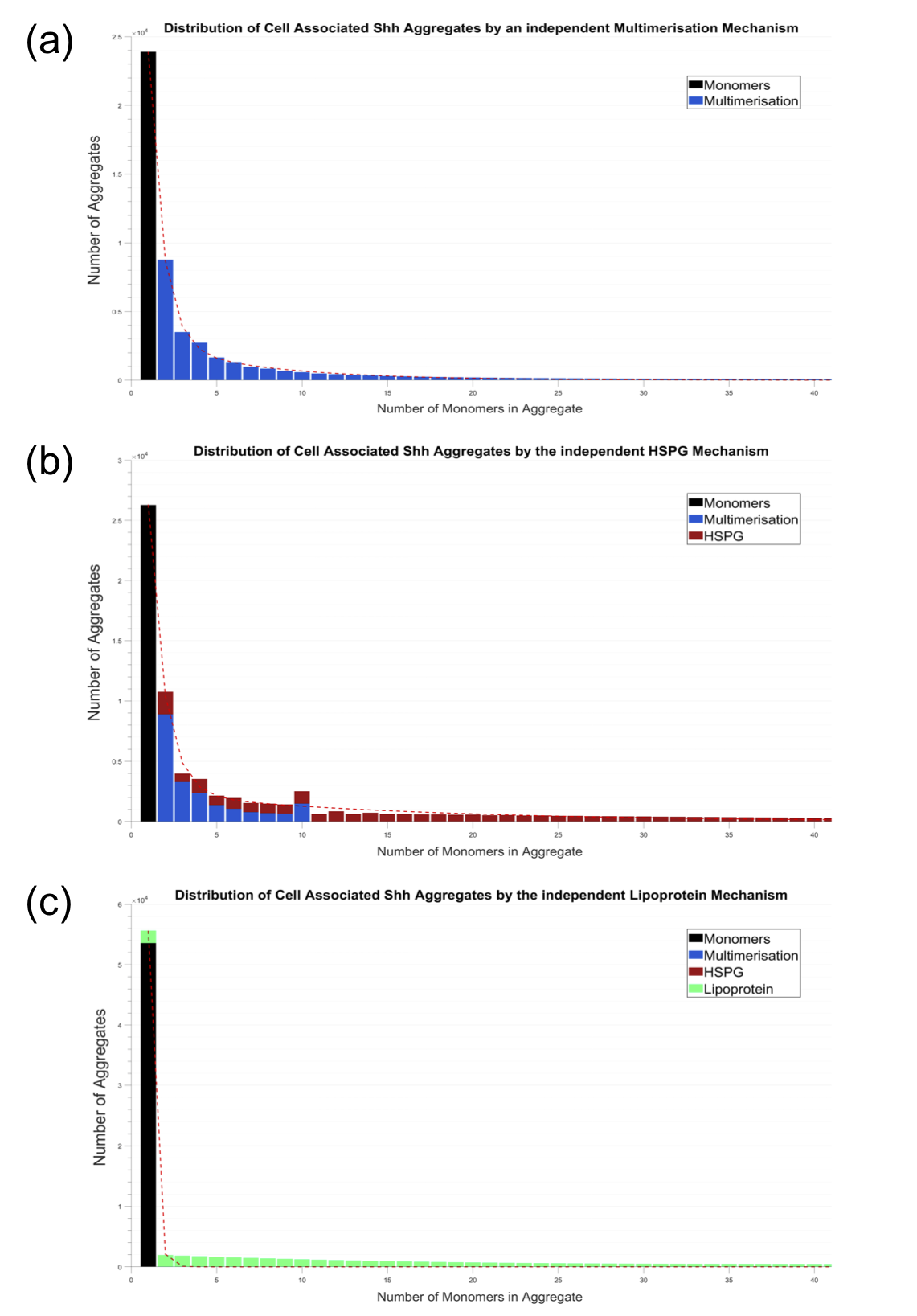

Supplement: S1 Fig — (TIFF) [file pcbi.1008562.s001.tiff]

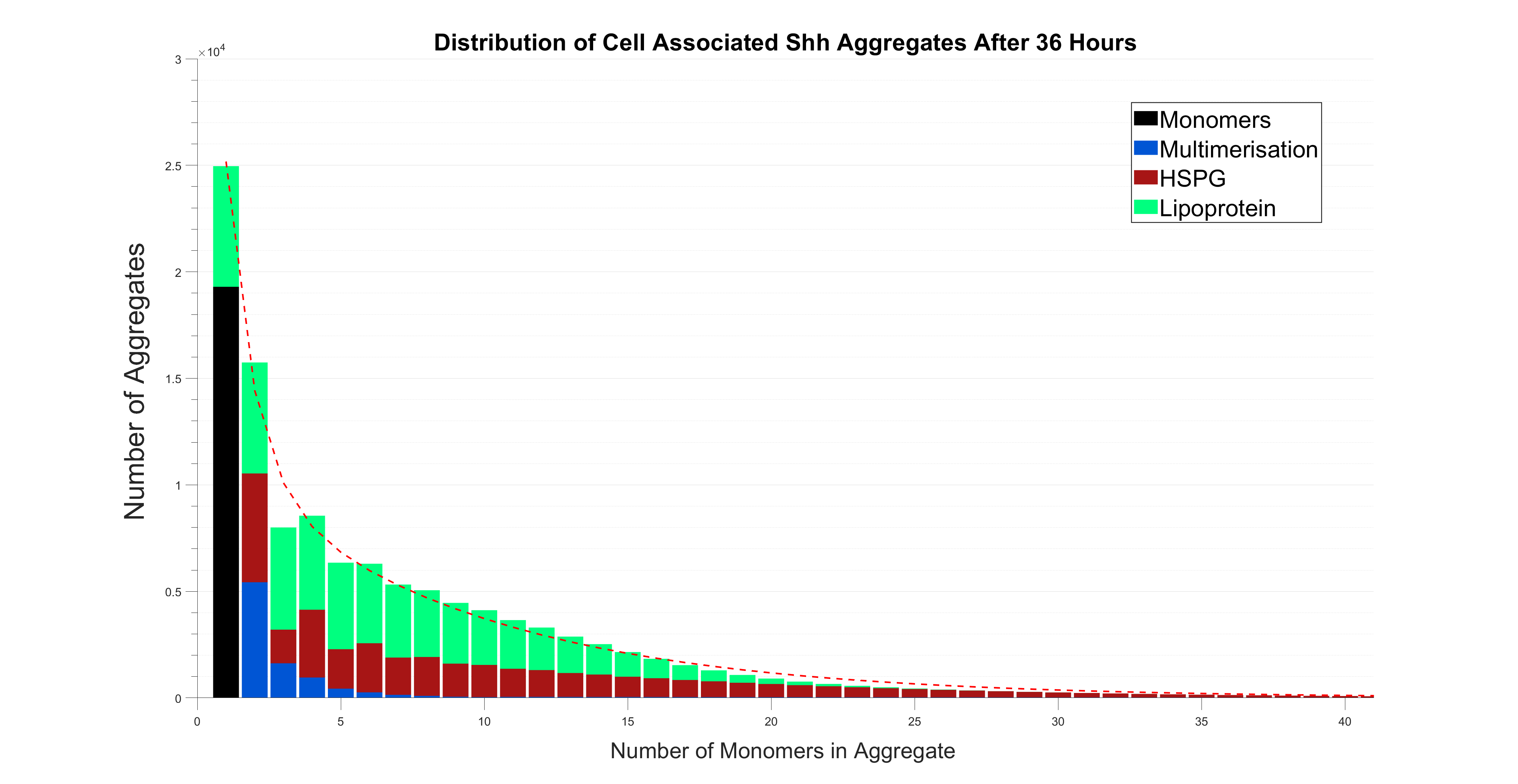

Supplement: S2 Fig — (TIF) [file pcbi.1008562.s002.tif]

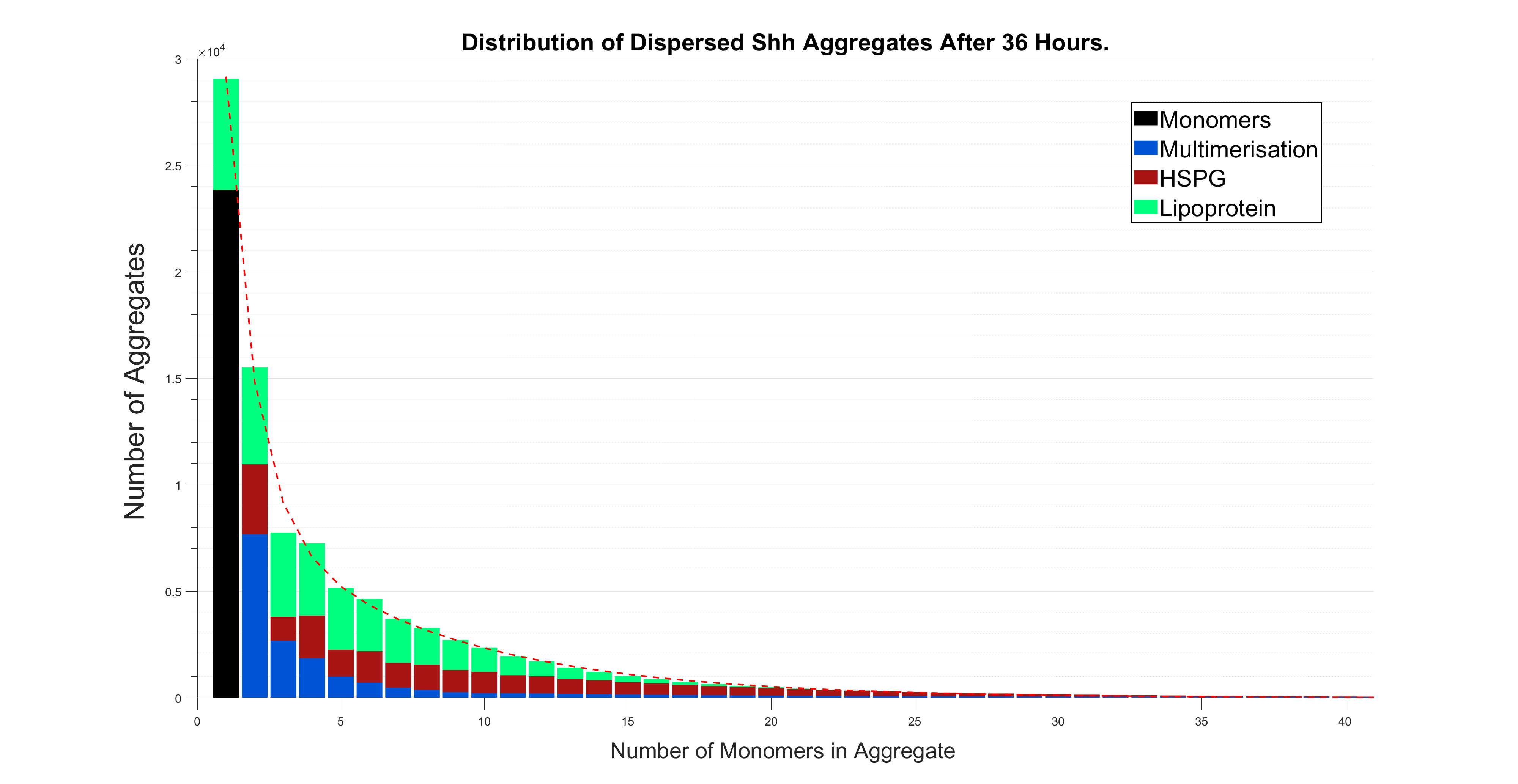

Supplement: S3 Fig — (TIF) [file pcbi.1008562.s003.tif]

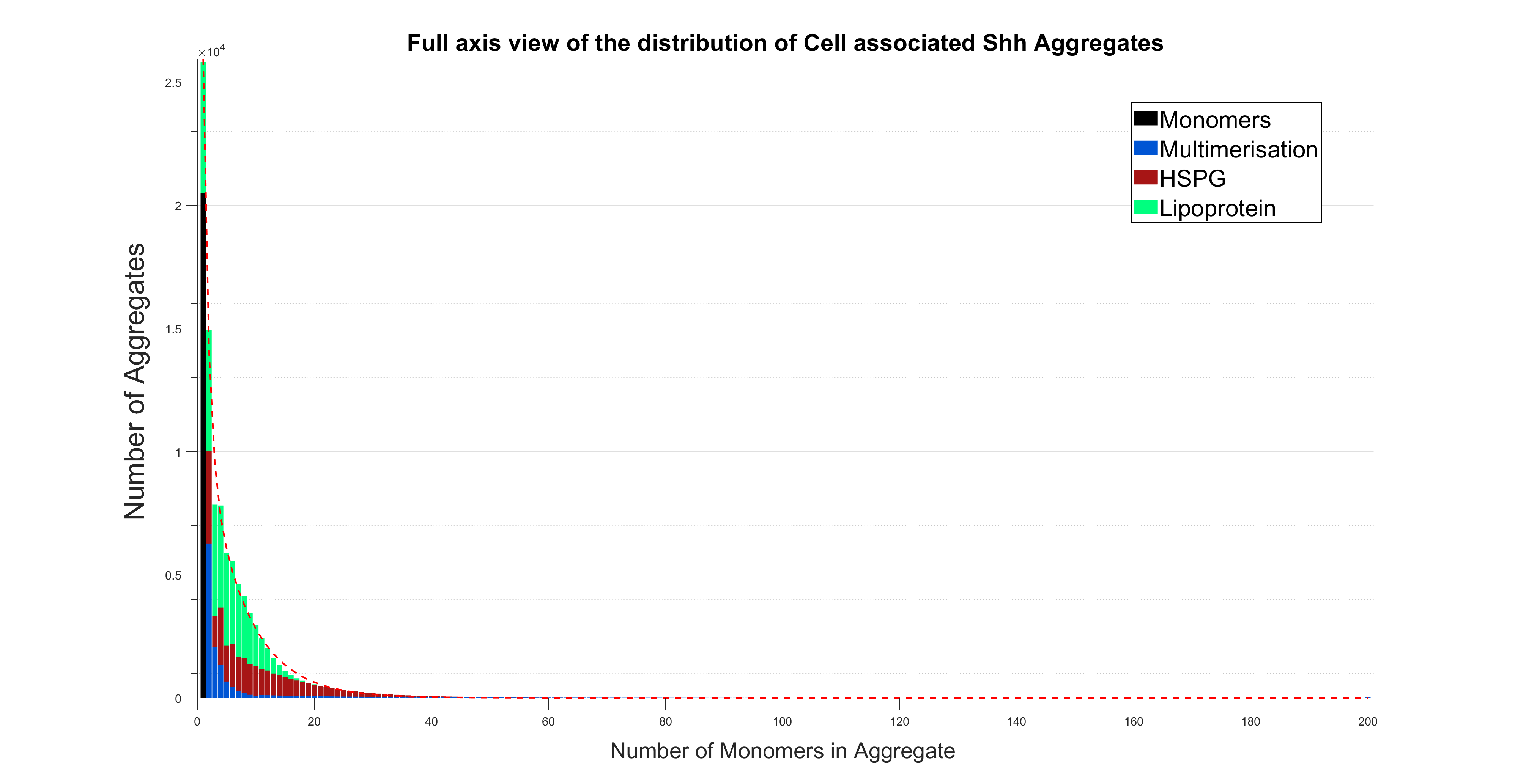

Supplement: S4 Fig — (TIF) [file pcbi.1008562.s004.tif]

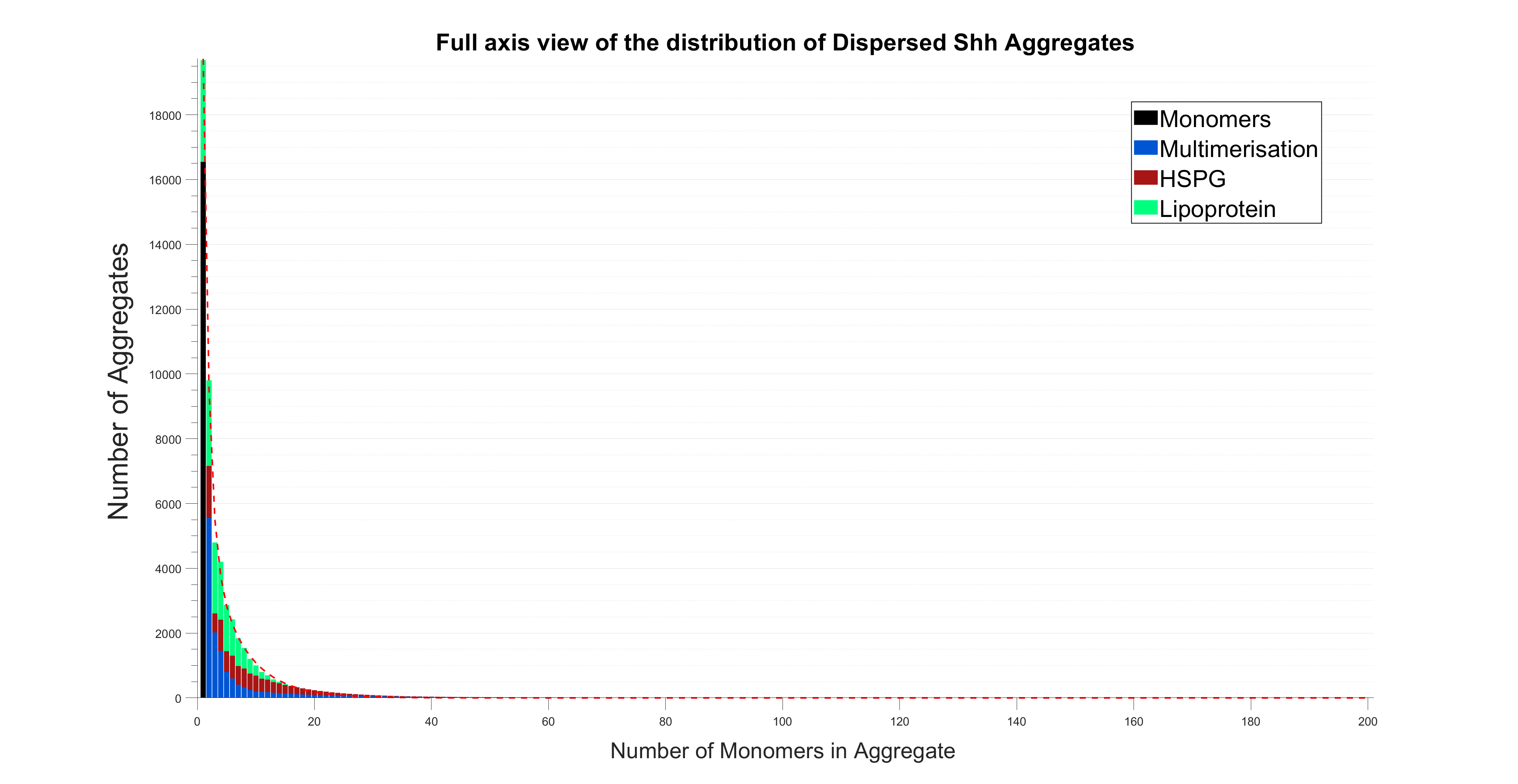

Supplement: S5 Fig — (TIF) [file pcbi.1008562.s005.tif]

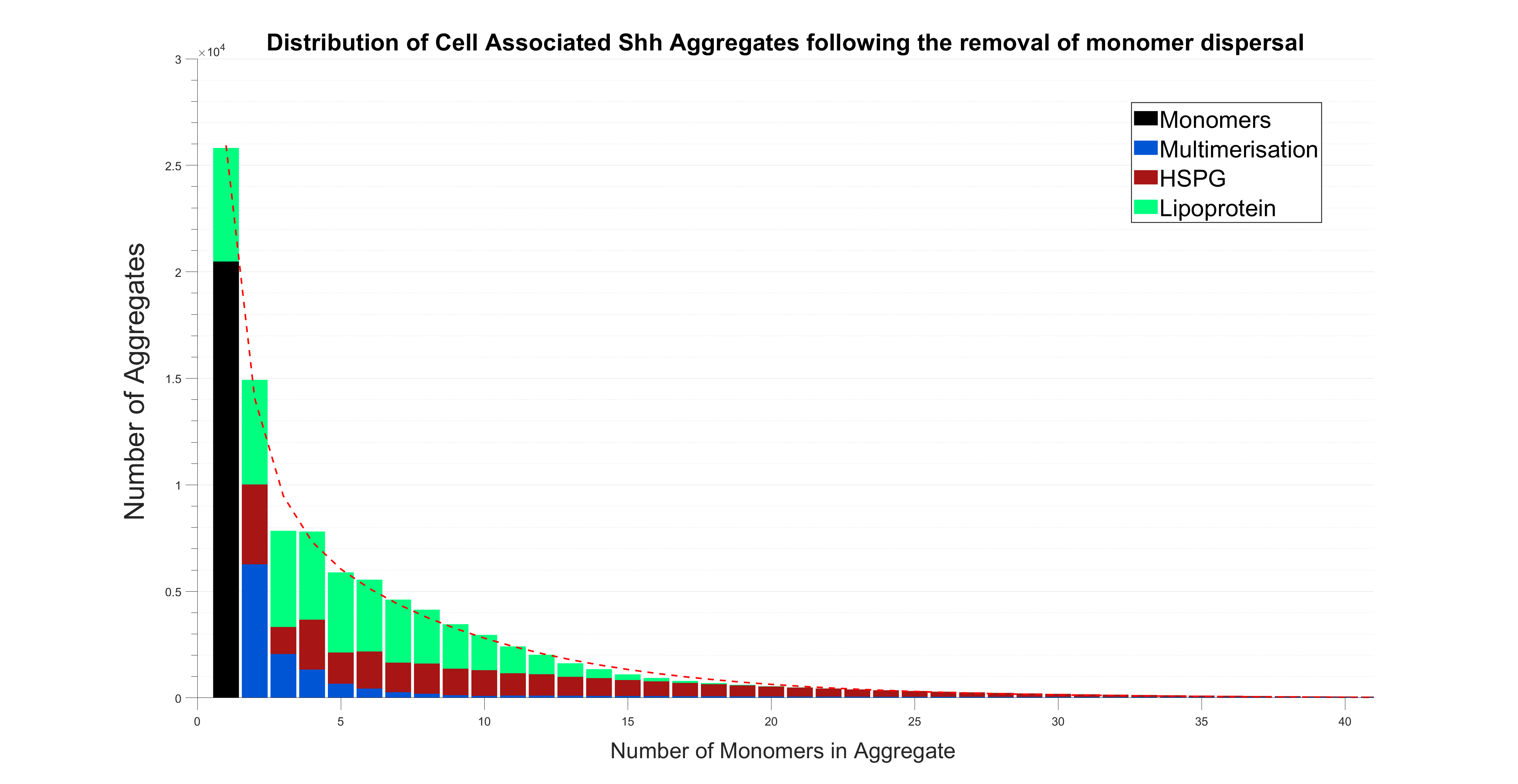

Supplement: S6 Fig — (TIF) [file pcbi.1008562.s006.tif]

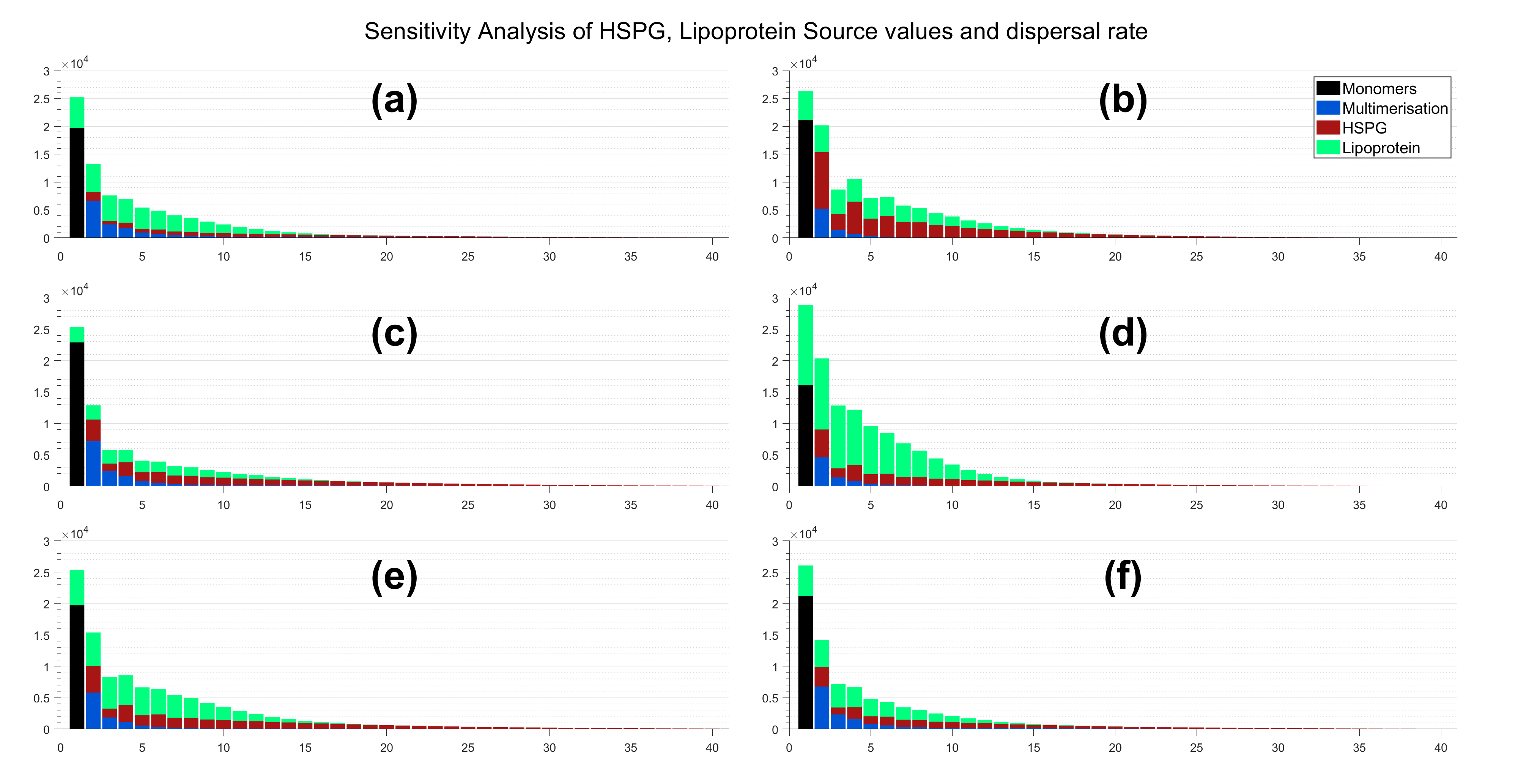

Supplement: S7 Fig — Shh aggregate distributions with: (a) half the rate of multimerisation; (b) doubled rate of multimerisation; (c) half the rate of HSPG binding; (d) doubled rate of HSPG binding; (e) half the rate of lipoprotein binding; and (f) double the rate of lipoprotein binding. (TIF) [file pcbi.1008562.s007.tif]

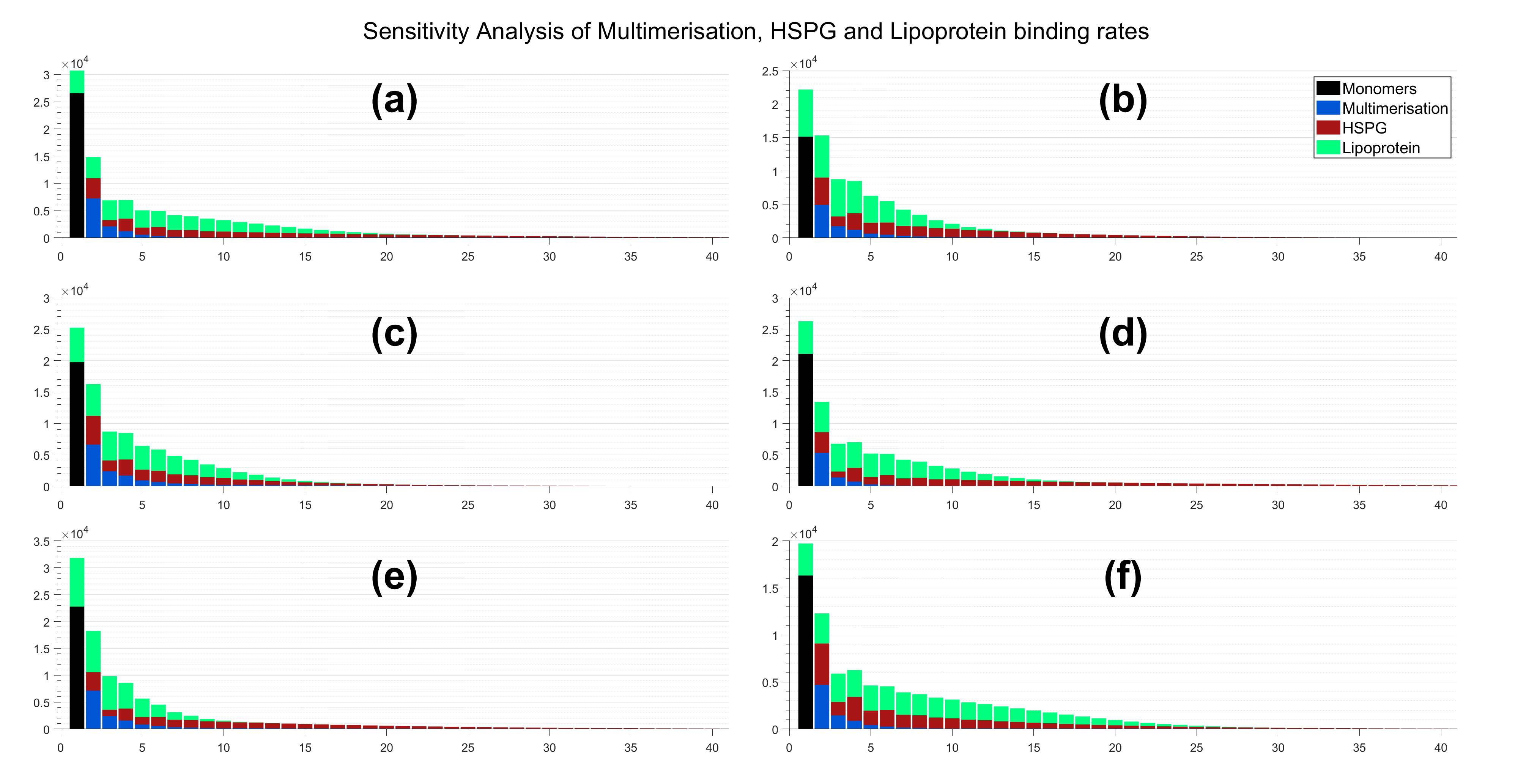

Supplement: S8 Fig — Shh aggregate distributions with: (a) half the rate of multimerisation; (b) doubled rate of multimerisation; (c) half the rate of HSPG binding; (d) doubled rate of HSPG binding; (e) half the rate of lipoprotein binding; and (f) double the rate of lipoprotein binding. (TIF) [file pcbi.1008562.s008.tif]

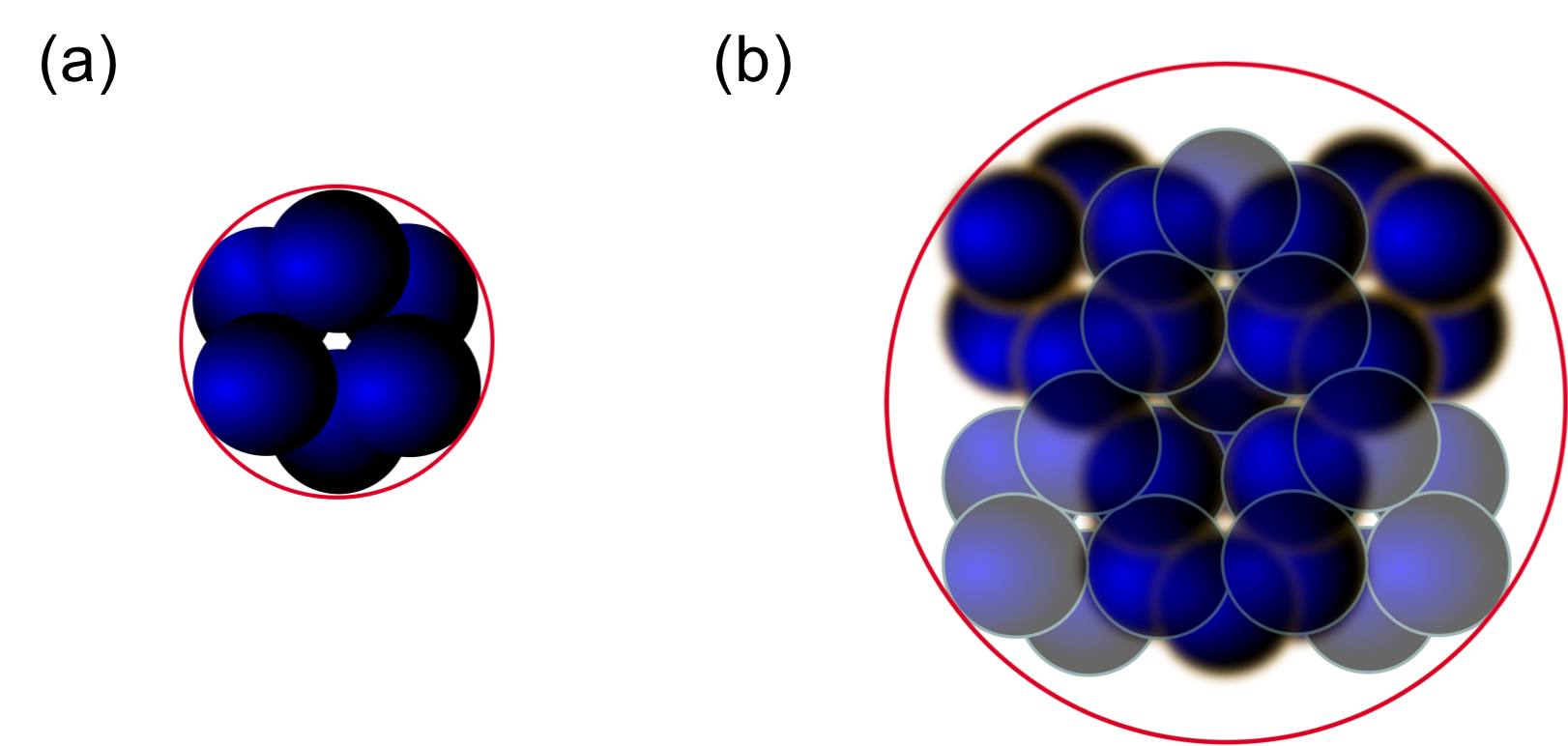

Supplement: S9 Fig — (TIFF) [file pcbi.1008562.s009.tiff]

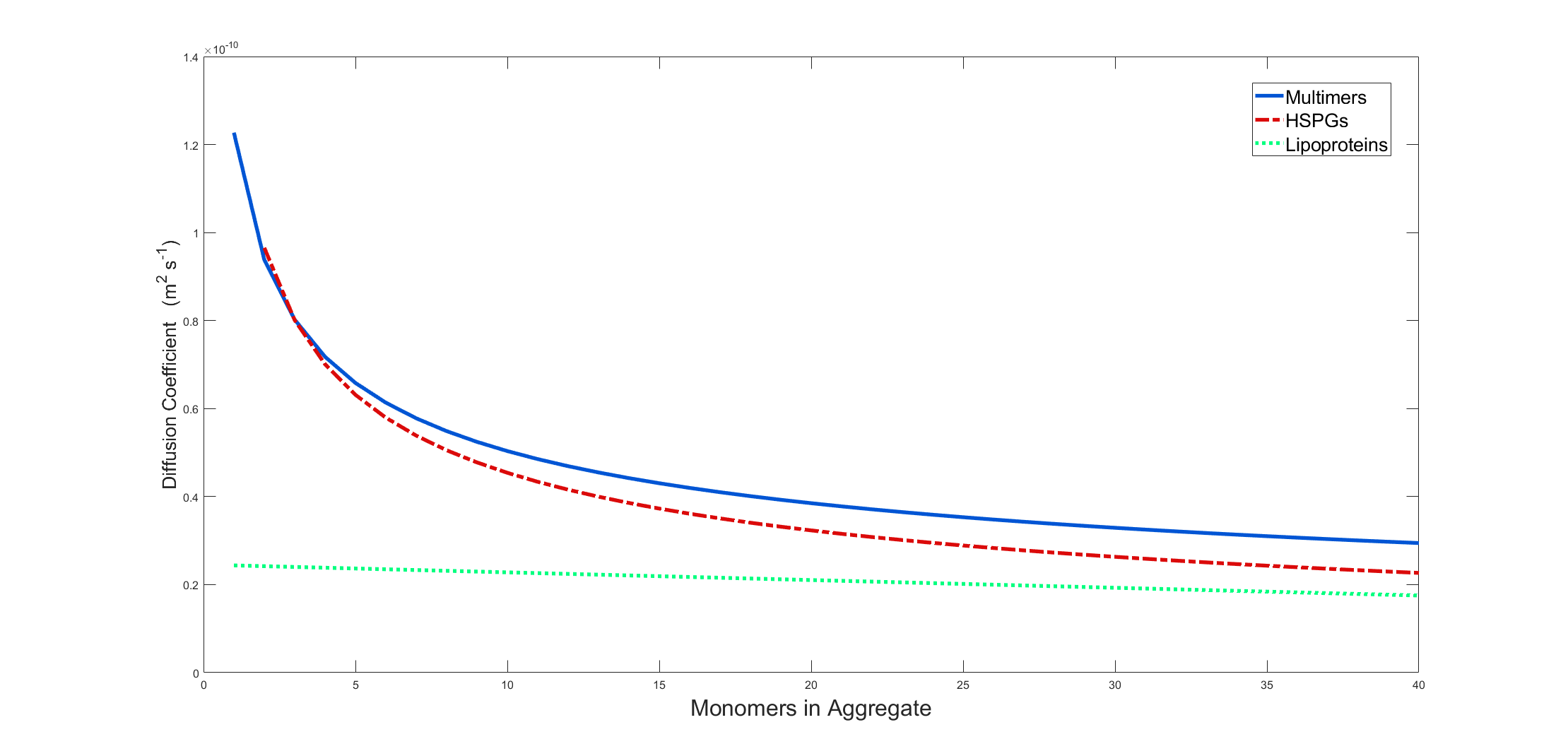

Supplement: S12 Fig — (TIF) [file pcbi.1008562.s012.tif]
