# Supplementary material for: A mathematical model of the role of aggregation in sonic hedgehog signalling
Source: PLoS Comput Biol. 2021 Feb 22;17(2):e1008562. doi: 10.1371/journal.pcbi.1008562 (PMC7932509; doi:10.1371/journal.pcbi.1008562)
Supplement: S1 Text — (PDF) [file pcbi.1008562.s013.pdf]

A Mathematical Approach to Understanding the Role of Aggregation in Sonic Hedgehog Signalling

Supplementary Information

Daniel J. A. Derrick, Kathryn Wolton, Richard Currie and Marcus John Tindall

S1 Model derivation

Our models describe the quantity of each potential aggregate size and their interactions in the formation of multimers. For the model equations described within this section we are required to set a 'largest' aggregate that can be formed. This aggregate is considered to consisting of n Shh proteins, where n is either given or is left arbitrary. We form nonlinear ordinary differential equations (ODEs) utilising the law of mass action.

S1.1 Multimerisation

We assume that multimerisation interactions occur in a pairwise manner; that is, multimers are formed via single interactions between multimers, monomers, or multimers and monomers. We do this as we consider the decreased likelihood of more than two Shh proteins, individually or as part of an aggregate, interacting within a local spatial location. Instead, we make the assumption that the concurrent interaction of multiple Shh aggregates and monomers can be viewed as a sequence of rapid, separate pairwise interactions.

An example is given below for a multimerisation system when $n = 3$ and as such is restricted to forming aggregates with as many as three Shh proteins to a single multimer. The nonlinear ODEs describing this process are given by Equation (1),

$$\frac{dx_1}{dt} = \overbrace{\alpha}^{\text{Source of monomers}} \underbrace{-2m_{1,1}x_1^2}_{\text{Formation of dimer}} \overbrace{-m_{1,2}x_1x_2}^{\text{Formation of trimer}} \underbrace{-\beta x_1}_{\text{Dispersal Term}}, \tag{1a}$$

$$\frac{dx_2}{dt} = m_{1,1}x_1^2 - m_{1,2}x_1x_2 - \beta x_2, \tag{1b}$$

$$\frac{dx_3}{dt} = m_{1,2}x_1x_2 - \beta x_3, \tag{1c}$$

where the initial conditions are given by

$$x_1(0) = 0, \qquad x_2(0) = 0, \quad \text{and} \quad x_3(0) = 0.$$

Equation (1) describes the rate of change in quantity of each size multimer. The monomer density, represented by $x_1 = x_1(t)$, increases with a constant rate α and represents an influx of Shh to the cell surface. The subsequent terms describe the removal of two monomers in the formation of a dimer and a single monomer in the formation of a trimer. The final terms of each equation describe the quantity-dependent dispersal of each monomer and multimer.

In the case of aggregates formed via multimerisation which are able to consist of up to n Shh protein, the governing equations are given by

$$\frac{dx_1}{dt} = \overbrace{\alpha}^{\text{Source of monomers}} \underbrace{- \sum_{l=1}^{n-1} m^*_{1,l} x_1 x_l}_{\text{Monomer interactions with multimers}} \overbrace{-\beta x_1}^{\text{Dispersal Term}}, \tag{2a}$$

$$\frac{dx_i}{dt} = \overbrace{\sum_{a \geq b > 0}^{a+b=i} m_{a,b} x_a x_b}^{\text{All combinations that form a multimer with } i \text{ Shh}} \underbrace{- \sum_{l=1}^{n-i} m^*_{i,l} x_i x_l}_{\text{Formation of larger multimers from multimer with } i \text{ Shh}} \overbrace{-\beta x_i}^{\text{Dispersal Term}}, \qquad \text{for } i = 2 : n, \tag{2b}$$

where the initial conditions are

$$x_l(0) = 0 \quad \text{for } l \in [1, n].$$

In Equation (2) we have the term $m^*_{a,b} = 2 \times m_{a,b}$ when $a = b$, and otherwise if $a \neq b$ we have $m^*_{a,b} = m_{a,b}$. As previously defined, binding rates are given by $m_{a,b}$ and describe the binding affinity for the interaction between a multimer (monomer if $a = 1$) with a associated monomers and a multimer with b (monomer if $b = 1$). We set the initial amount of Shh monomers and all aggregates (x_l for $l \in [1, n]$) to be equal to zero to represent the initial inactivity of Shh aggregation by the single cell we describe.

S1.2 Heparan Sulfate Proteoglycans

We assume that Shh-HSPG interactions occur in the following manner. Previously it was proposed that HSPGs would fulfill the role of acting as a 'scaffold' structure to promote the formation of large Shh aggregates. The work of Vyas and colleagues [1] in *D. melanogaster* suggests that Hh proteins undergo mandatory organisation events prior to interaction with HSPGs. We therefore made the assumption that Shh would be required to form

multimers to bind HSPGs and. The publication by Vyas et al. notes that the disruption of multimerisation interactions leads to their inability to bind with HSPGs; to emulate this we do not allow monomeric Shh to bind HSPGs. Further, to explore the role of HSPGs in the formation of large aggregates and its function as a scaffold, we limit the size to which multimers are able to bind the structures for these interactions. The length of heparin chains may be one such factor that restricts the size of multimers that binds as is suggested by the structural insights reported by Whalen and colleagues [2].

We model HSPGs with a population of 'free' particles which bind with Shh multimers to form aggregates. For this, Shh is recruited to HSPGs as multimers to increase the size of the aggregate. To represent this mechanism we are required to include a system for the formation of small multimers with which HSPGs interact. Below we show the nonlinear ODE model describing the formation of multimers with as many as three Shh monomers which are recruited by HSPGs to form aggregates with as many as 4 Shh proteins. This is represented in Equation 3.

$$\frac{dx_1}{dt} = \overbrace{\alpha}^{\substack{\text{Source of} \\ \text{Shh monomers}}} \underbrace{-2m_{1,1}x_1^2 - m_{1,2}x_1x_2}_{\text{Multimerisation terms}} \overbrace{-\beta x_1}^{\text{Monomer dispersal}}, \tag{3a}$$

$$\frac{dx_2}{dt} = m_{1,1}x_1^2 - m_{1,2}x_1x_2 \underbrace{-h_0x_2H_0}_{\substack{\text{Dimers recruited} \\ \text{to HSPG aggregates}}} -\beta x_2, \tag{3b}$$

$$\frac{dx_3}{dt} = m_{1,2}x_1x_2 - h_0x_3H_0 - \beta x_3, \tag{3c}$$

$$\frac{dH_0}{dt} = \overbrace{\delta}^{\substack{\text{Source of} \\ \text{free HSPGs}}} \overbrace{-h_0x_2H_0 - h_0x_3H_0}^{\substack{\text{Multimers binding} \\ \text{to free HSPGs}}}, \tag{3d}$$

$$\frac{dH_2}{dt} = \underbrace{h_0x_2H_0}_{\substack{\text{HSPG aggregate} \\ \text{formation}}} -h_2x_2H_2 \underbrace{-\beta H_2}_{\substack{\text{HSPG aggregate} \\ \text{dispersal}}}, \tag{3e}$$

$$\frac{dH_3}{dt} = h_0x_3H_0 - \beta H_3, \tag{3f}$$

$$\frac{dH_4}{dt} = h_2x_2H_2 - \beta H_4, \tag{3g}$$

where the initial conditions are given by

$$x_a(0) = 0 \quad \text{for} \quad a = 1:3 \quad \text{and} \quad H_a(0) = 0 \quad \text{for} \quad a = 0:4.$$

In the HSPG model the included multimerisation system is as previously described, with x_i representing the population of multimers that consist of i Shh and x_1 describing the quantity of monomers. Analogously, we represent the population of HSPGs with i Shh

bound by H_i, meaning H_0 represents those that are unoccupied by Shh. Of note, H_1 is not modelled as monomers are not able to bind HSPGs. The constant rate of dispersal is described by β and remaining terms are as discussed in Equations (3).

We next consider the derivation of a HSPG mechanism that is able to produce aggregates with as many as n bound. For this we include a system of Shh multimerisation to demonstrate interactions with HSPGs. Whilst we later elect a largest size for the constructed multimers based on literature, in this example we set the maximum multimer size to be arbitrary and given by p.

The system of nonlinear ODEs that describe the HSPG production of aggregates with up to n Shh monomers is given by

$$\frac{dx_1}{dt} = \overbrace{\alpha}^{\text{Monomer source}} \underbrace{- \sum_{l=1}^{p-1} m^*_{(1,l)} x_1 x_l}_{\substack{\text{Monomers bind to multimers} \\ \text{consisting of as many as } p-1 \text{ Shh}}} \overbrace{-\beta x_1}^{\text{Monomer dispersal}}, \tag{4a}$$

$$\frac{dx_i}{dt} = \underbrace{\sum_{j \geq k > 0}^{j+k=i} m_{(j,k)} x_j x_k}_{\substack{\text{Formation of multimers} \\ \text{consisting of } i \text{ Shh}}} - \sum_{l=i}^{p-i} m^*_{(i,l)} x_i x_l \underbrace{-h_0 x_i H_0}_{\substack{\text{Multimer binds with} \\ \text{free HSPGs}}} \tag{4b}$$

$$\underbrace{- \sum_{c=2}^{n-i} h_c x_i H_c}_{\substack{\text{Multimer binds with} \\ \text{HSPG aggregate}}} \underbrace{-\beta x_i}_{\text{Multimer dispersal}}, \qquad \text{for } i = 2:p,$$

$$\frac{dH_0}{dt} = \overbrace{\delta}^{\text{HSPG source}} \overbrace{- \sum_{i=2}^{p} h_0 x_i H_0}^{\substack{\text{Multimers binding} \\ \text{to free HSPGs}}}, \tag{4c}$$

$$\frac{dH_i}{dt} = \underbrace{h_0 x_i H_0}_{\substack{\text{Formation of HSPG} \\ \text{aggregates from free HSPGs}}} + \sum_{c \geq 2,\ p \geq d \geq 2}^{c+d=i} h_c x_d H_c \tag{4d}$$

$$- \sum_{d=2}^{\min(n-i,p)} h_i x_d H_i \underbrace{-\beta H_i}_{\substack{\text{HSPG aggregate} \\ \text{dispersal}}}, \qquad \text{for } i = 2:p, \tag{4e}$$

$$\frac{dH_j}{dt} = \underbrace{\sum_{c\geq 2,\ p\geq d\geq 2}^{c+d=j} h_c x_d H_c}_{\substack{\text{If the size of HSPG aggregates exceed that of multimers,} \\ \text{they cannot be formed by multimers binding to free HSPGs}}} - \sum_{d=2}^{min(n-j,p)} h_j x_d H_j - \beta H_j, \qquad \text{for } j = p+1 : n, \tag{5a}$$

where the initial conditions are given by

$$x_a(0) = 0, \quad \text{for} \quad a = 1 : p,$$

and

$$H_a(0) = 0, \quad \text{for} \quad a = 0 : n.$$

Terms shown in Equation (5) are as defined in previous subsections. This includes the term $m^*_{a,b}$, which is defined by $m^*_{a,b} = 2 \times m_{a,b}$ when $a = b$, and otherwise if $a \neq b$ we have $m^*_{a,b} = m_{a,b}$.

S1.3 Lipoproteins

We model lipoprotein interactions similar to that of HSPGs in that particles act as a surface with which Shh binds in the formation of aggregates. Currently, the understanding of lipoprotein-Shh interactions remains mostly unclear and there is limited experimental evidence that indicates how the mechanism may operate. We therefore make a number of assumptions based on how we interpret these events to occur. A central assumption we make is that Shh will be recruited to and binds lipoproteins in singular interactions. This means we make the presumption that Shh cannot bind lipoproteins after forming into a multimer. We make this distinction as some researchers have suggested that Shh utilises its lipid heads to bind the phospholid monolayer of lipoproteins [3]; the formation of multimers would most likely occur such that Shh directs its lipid heads into the core, which would negate the potential to associate with lipoproteins. In addition, the formation of multimers would, as is hypothesised in previous literature discussions [4, 5], resolve Shh of its hydrophobicity without any requirement of lipoprotein interaction. In a preliminary example we describe the formation of lipoprotein aggregates that bind as many as three Shh monomers. This is represented by the system of nonlinear ODEs given in Equation (6),

$$\frac{dx_1}{dt} = \overbrace{\alpha}^{\text{Monomer source}} \underbrace{-k_1 x_1 l_0}_{\text{Monomers bind to free lipoproteins}} \overbrace{-k_2 x_1 l_1 - k_3 x_1 l_2}^{\text{Monomers bind lipoprotein aggregates}} \underbrace{-\beta x_1}_{\text{Monomer dispersal}}, \tag{6a}$$

$$\frac{dl_0}{dt} = \overbrace{\gamma}^{\text{Lipoprotein source}} -k_1 x_1 l_0, \tag{6b}$$

$$\frac{dl_1}{dt} = k_1 x_1 l_0 - k_2 x_1 l_1 \overbrace{-\beta l_1}^{\text{Lipoprotein dispersal}}, \tag{6c}$$

$$\frac{dl_2}{dt} = k_2 x_1 l_1 - k_3 x_1 l_2 - \beta l_2, \tag{6d}$$

$$\frac{dl_3}{dt} = k_3 x_1 l_2 - \beta l_3, \tag{6e}$$

where the initial conditions are given by

$$x_1(0) = 0, \quad l_0(0) = 0, \quad l_1(0) = 0, \quad l_2(0) = 0 \quad \text{and} \quad l_3(0) = 0.$$

In the system of equations given by Equation (6) a number of terms are as previously defined in Equation (1); the constant addition of Shh monomers is represented by α and β describes the rate of dispersal of aggregates from the cell surface. In addition, we use γ to represent the constant addition of free lipoproteins that are not bound by Shh.

We next show the system of equations that describes the formation of lipoprotein aggregates that are able to bind as many as n Shh monomers. This is given by the following system of nonlinear ODEs,

$$\frac{dx_1}{dt} = \overbrace{\alpha}^{\text{Monomer source}} \underbrace{-k_1 x_1 l_0}_{\substack{\text{Monomer binding} \\ \text{to free lipoproteins}}} \overbrace{-\sum_{d=1}^{n} k_{d+1} x_1 l_d}^{\text{Monomers bind lipoprotein aggregates}} \underbrace{-\beta x_1}_{\text{Monomer dispersal}}, \tag{7a}$$

$$\frac{dl_0}{dt} = \overbrace{\gamma}^{\text{Lipoprotein source}} -k_1 l_0 x_1, \tag{7b}$$

$$\frac{dl_i}{dt} = \overbrace{k_i l_{i-1} x_1}^{\substack{\text{Formation of lipoprotein} \\ \text{aggregate with } i \text{ Shh bound}}} \underbrace{-k_{i+1} x_1 l_i}_{\substack{\text{Lipoprotein:Shh monomer} \\ \text{association}}} \overbrace{-\beta l_i}^{\substack{\text{Lipoprotein} \\ \text{aggregate dispersal}}}, \qquad \text{for } i = 1 : n-1, \tag{7c}$$

$$\frac{dl_n}{dt} = k_n x_1 l_{n-1} - \beta l_n, \tag{7d}$$

where the initial conditions are given by

$$x_1(0) = 0, \quad l_0(0) = 0 \quad \text{and} \quad l_a(0) = 0 \quad \text{for} \quad a = 1 : n.$$

References

1. Vyas N, Goswami D, Manonmani A, Sharma P, Ranganath Ha, Vijayraghavan K, et al. Nanoscale Organization of Hedgehog Is Essential for Long-Range Signaling. Cell. 2008;133(7):12141227. doi:10.1016/j.cell.2008.05.026.

2. Whalen DM, Malinauskas T, Gilbert RJC, Siebold C. Structural insights into proteoglycan-shaped Hedgehog signaling. PNAS. 2013;110(41):1642016425. doi:10.1073/pnas.1310097110.

3. Palm W, Swierczynska MM, Kumari V, Ehrhart-Bornstein M, Bornstein SR, Eaton S. Secretion and Signaling Activities of Lipoprotein-Associated Hedgehog and Non-Sterol-Modified Hedgehog in Flies and Mammals. PLoS Biology. 2013;11(3). doi:10.1371/journal.pbio.1001505.

4. Feng J, White B, Tyurina OV, Guner B, Larson T, Lee HY, et al. Synergistic and antagonistic roles of the Sonic hedgehog N- and C-terminal lipids. Development. 2004;131(17):4357–4370. doi:10.1242/dev.01301.

5. Li Y, Zhang H, Litingtung Y, Chiang C. Cholesterol modification restricts the spread of Shh gradient in the limb bud. Proceedings of the National Academy of Sciences. 2006;103(17):65486553. doi:10.1073/pnas.0600124103.
