# Supplementary material for: A mathematical model of the role of aggregation in sonic hedgehog signalling
Source: PLoS Comput Biol. 2021 Feb 22;17(2):e1008562. doi: 10.1371/journal.pcbi.1008562 (PMC7932509; doi:10.1371/journal.pcbi.1008562)
Supplement: S2 Text — (PDF) [file pcbi.1008562.s014.pdf]

A Mathematical Approach to Understanding the Role of Aggregation in Sonic Hedgehog Signalling

Supplementary Information

Daniel J. A. Derrick, Kathryn Wolton, Richard Currie and Marcus John Tindall

S2 Estimating parameter values

Currently, the amount of available data that would be essential to inform model parameters is largely insufficient. Nonetheless, we are able to effectively limit the parameter space by considering previous estimates made by other and qualitative data for aggregate production. We first note that as our model considers a single cell we make the initial assumption that 800 Shh are produced per minute, which follows from an estimate made by Dillon et al. for a Shh signalling model [1]. This gives the rate of monomer production per day (α) to be 1,152,000 proteins per day. Our remaining parameters were estimated such that each mechanism was appropriately represented and contributed in conformity with the literature. First, we assumed sensible upper and lower bounds for the expression of HSPGs and lipoproteins and we initially based this on the ratio of monomer to particle that is introduced over the 24-hour simulation period. We reason that the expression of a particular particle, either HSPG of lipoprotein, cannot not exceed that of Shh monomers. We found that an excessive rate of particle introduction would drive a particular mechanism to dominate aggregate production and leave the remaining mechanisms to become more redundant. On the other hand, it is equally important that the source of particles is not too low and mechanisms are able to provide a sufficient contribution to aggregate production, which would otherwise be in disagreement with literature that indicates each mechanism has an important role in aggregate production. We observed that if the rate of introduction for HSPG or lipoprotein particles was too low the aggregates produced via the relevant mechanisms would always be cell-associated in the largest sizes. We reason that this instance is largely unlikely to be biologically feasible and thus set a lower bound such that aggregates would be produced with a greater variation in the amount of Shh associated.

We considered it important to utilise values for the introduction of HSPGs and lipoproteins that would not greatly impact multimerisation and would also not excessively diminish

the cell-associated monomer population. We also initially chose to keep the binding rates of multimerisation, lipoproteins and HSPGs within a similar order of magnitude and with values that were indicated by the data fitting results. We obtained a range of values for the rate of aggregate dispersal by data fitting both the cell-associated and dispersal data reported by [2] simultaneously, but noted the range of values was broad. We found that varying the rate of aggregate dispersal does not significantly impact the rate of aggregate production and the distribution of cell-associate aggregates is not visibly affected unless it is almost 100-fold larger.

We completed a sensitivity analysis to refine our values further and again qualitatively compared our simulation results at 24 hours with that of Koleva and colleagues [2]. The parameter values we used are given in S1 Table.

Parameter	Definition	Value	Source
α	Source of monomers	1.152×10^6 monomers/day	[1]
δ	Source of HSPGs	4.60×10^4 particles/day	Estimate.
γ	Source of lipoproteins	6.00×10^4 particles/day	Estimate.
β	Rate of dispersal	0.75 day^{-1}	Estimate.
$m_{a,b}$	Multimerisation binding rate	6.00×10^{-4} (multimer day)$^{-1}$	Estimate.
h_i	Multimer to HSPG binding rate	4.00×10^{-4} (multimer day)$^{-1}$	Estimate.
k_i	Monomer to lipoprotein binding rate	5.00×10^{-4} (protein day)$^{-1}$	Estimate.

S1 Table: Parameters for the aggregation model.

We reason that our chosen values are suitable for each mechanism. The source of HSPGs in respect to monomer availability means that the particle to monomer ratio is ∼25:1. Larger sources of HSPGs were found to be more disruptive to multimerisation due to a greater rate of recruitment. For our lipoprotein source value, we note that that the ratio for the expression of lipoproteins to monomers is 19.2:1 This expression allows the lipoprotein to contribute a reasonable degree of competition for monomers with the multimerisation mechanism, but does not cause lipoproteins to excessively dominate aggregate production.

References

1. Dillon R, Gadgil C, Othmer HG. Short- and long-range effects of Sonic hedgehog in limb development. Proceedings of the National Academy of Sciences of the United States of America. 2003;100:10152–10157. doi:10.1073/pnas.1830500100.

2. Koleva MV, Rothery S, Spitaler M, Neil MAA, Magee AI. Sonic hedgehog multimerization: A self-organizing event driven by post-translational modifications? Molecular Membrane Biology. 2015;32(3):6574. doi:10.3109/09687688.2015.1066895.
