# Supplementary material for: A mathematical model of the role of aggregation in sonic hedgehog signalling
Source: PLoS Comput Biol. 2021 Feb 22;17(2):e1008562. doi: 10.1371/journal.pcbi.1008562 (PMC7932509; doi:10.1371/journal.pcbi.1008562)
Supplement: S3 Text — (PDF) [file pcbi.1008562.s015.pdf]

A Mathematical Approach to Understanding the Role of Aggregation in Sonic Hedgehog Signalling

Supplementary Information

Daniel J. A. Derrick, Kathryn Wolton, Richard Currie and Marcus John Tindall

S3 Individual mechanism distributions

We investigated the distribution at 24-hours when each mechanism functions independent of the others. Using the parameters described in S1 Table we simulate aggregate formation whilst opposing mechanism interactions are disabled, with the exception of HSPGs which requires a system for the formation of small multimers (as many as 10 monomers to an aggregate) with which it binds. The distributes produced are shown in S1 Fig.

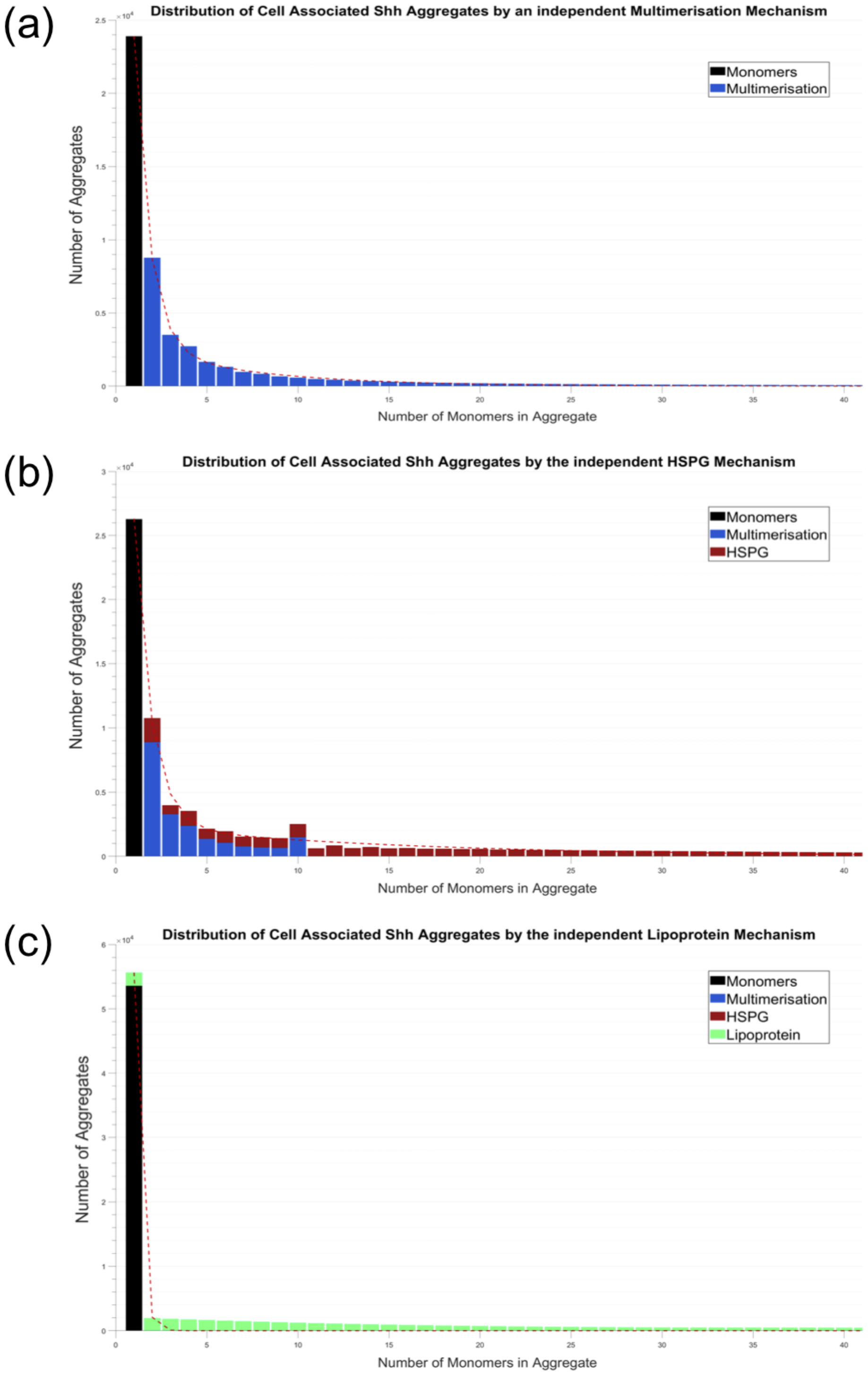

S1 Fig: Shh aggregate formation as a result of each individual mechanism: Here, aggregates are formed via **(a)** multimerisation, **(b)** HSPG and **(c)** lipoprotein recruitment in absence of the remaining two mechanisms. We allow the formation of small multimers in the individual HSPG model to allow recruitment to occur. Multimerisation in this model is however restricted and cannot consist of more than 10 Shh monomers. Simulations shown are at 24 hours.
