# Supplementary material for: A mathematical model of the role of aggregation in sonic hedgehog signalling
Source: PLoS Comput Biol. 2021 Feb 22;17(2):e1008562. doi: 10.1371/journal.pcbi.1008562 (PMC7932509; doi:10.1371/journal.pcbi.1008562)
Supplement: S4 Text — (PDF) [file pcbi.1008562.s016.pdf]

A Mathematical Approach to Understanding the Role of Aggregation in Sonic Hedgehog Signalling

Supplementary Information

Daniel J. A. Derrick, Kathryn Wolton, Richard Currie and Marcus John Tindall

S4 Steady-state distribution

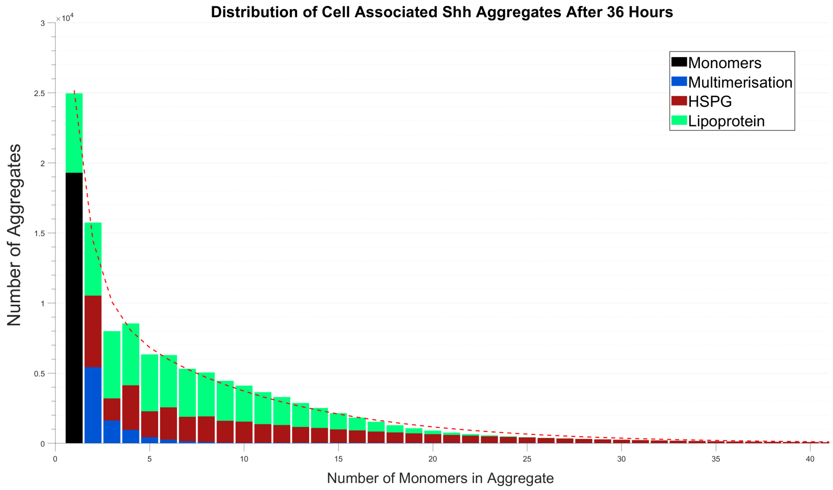

S2 Fig: Cell associated Shh distribution at steady state: Shh aggregate formation as a result of multimerisation, HSPG and lipoprotein association: Tables indicate the percentage of each mechanism responsible for forming the respective size aggregate in terms of the total number of Shh protein monomers and aggregates formed. Simulation shown is at steady state ($\sim$36 hours).

	1	2	3	4	5	6	7	8	9	10	
Monomers	16.31	-	-	-	-	-	-	-	-	-	
Multimers	-	4.59	1.37	0.81	0.37	0.22	0.12	0.08	0.05	0.03	
HSPGs	0.00	4.32	1.34	2.69	1.57	1.95	1.48	1.54	1.31	1.28	
Lipoproteins	4.79	4.41	4.05	3.73	3.43	3.16	2.90	2.65	2.41	2.17	
	11	12	13	14	15	16	17	18	19	20	
Monomers	-	-	-	-	-	-	-	-	-	-	
Multimers	0.04	0.04	0.04	0.04	0.04	0.04	0.03	0.03	0.03	0.03	
HSPGs	1.12	1.06	0.95	0.89	0.80	0.75	0.68	0.63	0.57	0.52	
Lipoproteins	1.93	1.69	1.44	1.21	0.98	0.77	0.59	0.43	0.31	0.21	
	21	22	23	24	25	26	27	28	29	30	
Monomers	-	-	-	-	-	-	-	-	-	-	
Multimers	0.03	0.03	0.03	0.03	0.03	0.02	0.02	0.02	0.02	0.02	
HSPGs	0.48	0.44	0.40	0.36	0.33	0.30	0.27	0.24	0.22	0.20	
Lipoproteins	0.14	0.09	0.06	0.03	0.02	0.01	0.01	0.00	0.00	0.00	
	31	32	33	34	35	36	37	38	39	40	> 40
Monomers	-	-	-	-	-	-	-	-	-	-	-
Multimers	0.02	0.02	0.02	0.02	0.02	0.02	0.02	0.02	0.02	0.02	1.52
HSPGs	0.18	0.16	0.14	0.12	0.11	0.10	0.09	0.08	0.07	0.06	0.36
Lipoproteins	0.00	0.00	0.00	0.00	0.00	0.00	0.00	0.00	0.00	0.00	0.00

S2 Table: Percentage breakdown of mechanisms that form the steady-state cell associated Shh aggregate distribution as shown in S2 Fig. The percentage of each mechanism responsible for forming the respective size aggregate in terms of the total number of Shh protein monomers and aggregates formed.

	1	2	3	4	5	6	7	8	9	10	
Monomers	24.33	-	-	-	-	-	-	-	-	-	
Multimers	-	7.84	2.74	1.90	1.02	0.73	0.49	0.39	0.28	0.22	
HSPGs	0.00	3.35	1.15	2.05	1.28	1.50	1.19	1.21	1.06	1.02	
Lipoproteins	5.34	4.65	4.03	3.47	2.97	2.52	2.11	1.75	1.44	1.16	

	11	12	13	14	15	16	17	18	19	20	
Monomers	-	-	-	-	-	-	-	-	-	-	
Multimers	0.22	0.21	0.19	0.18	0.16	0.15	0.14	0.13	0.12	0.11	
HSPGs	0.86	0.83	0.72	0.67	0.60	0.55	0.49	0.44	0.40	0.36	
Lipoproteins	0.92	0.71	0.54	0.40	0.29	0.21	0.14	0.09	0.06	0.04	

	21	22	23	24	25	26	27	28	29	30	
Monomers	-	-	-	-	-	-	-	-	-	-	
Multimers	0.10	0.10	0.09	0.09	0.08	0.08	0.07	0.07	0.07	0.06	
HSPGs	0.32	0.29	0.25	0.23	0.20	0.18	0.16	0.14	0.12	0.11	
Lipoproteins	0.02	0.01	0.01	0.00	0.00	0.00	0.00	0.00	0.00	0.00	

	31	32	33	34	35	36	37	38	39	40	>40
Monomers	-	-	-	-	-	-	-	-	-	-	-
Multimers	0.06	0.06	0.06	0.05	0.05	0.05	0.05	0.05	0.04	0.04	1.82
HSPGs	0.09	0.08	0.07	0.06	0.05	0.05	0.04	0.03	0.03	0.03	0.14
Lipoproteins	0.00	0.00	0.00	0.00	0.00	0.00	0.00	0.00	0.00	0.00	0.00

S3 Table: Percentage breakdown of mechanisms that form the steady-state dispersed Shh aggregate distribution as shown in S3 Fig. The percentage of each mechanism responsible for forming the respective size aggregate in terms of the total number of Shh protein monomers and aggregates formed.

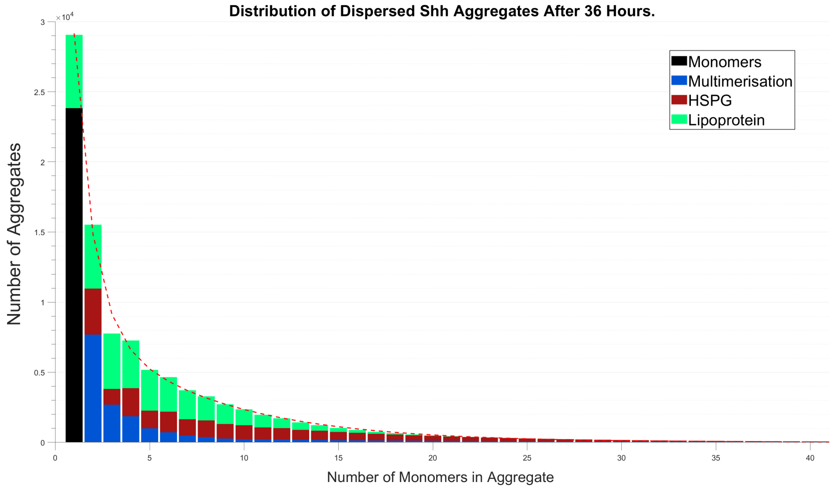

S3 Fig: Dispersed Shh distribution at steady state: Shh aggregate formation as a result of multimerisation, HSPG and lipoprotein association: Tables indicate the percentage of each mechanism responsible for forming the respective size aggregate in terms of the total number of Shh protein monomers and aggregates formed. Simulation shown is at steady state (∼36 hours).
