# Supplementary material for: A mathematical model of the role of aggregation in sonic hedgehog signalling
Source: PLoS Comput Biol. 2021 Feb 22;17(2):e1008562. doi: 10.1371/journal.pcbi.1008562 (PMC7932509; doi:10.1371/journal.pcbi.1008562)
Supplement: S5 Text — (PDF) [file pcbi.1008562.s017.pdf]

A Mathematical Approach to Understanding the Role of Aggregation in Sonic Hedgehog Signalling

Supplementary Information

Daniel J. A. Derrick, Kathryn Wolton, Richard Currie and Marcus John Tindall

S5 Full view of distribution figure axis

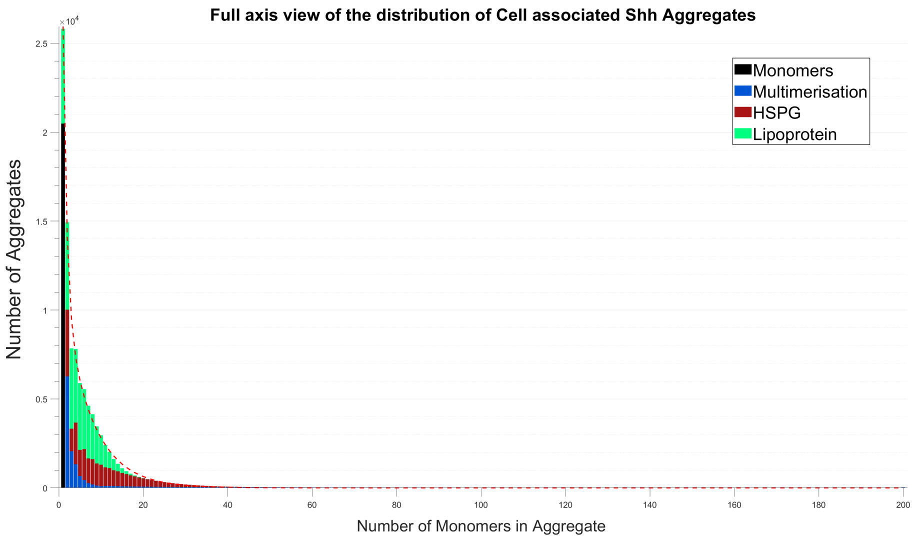

S4 Fig: Full axis view of the Cell Associated Shh distribution. Figures show the full distribution of aggregates that consist of up to 200 monomers. Simulation shown is at 24 hours.

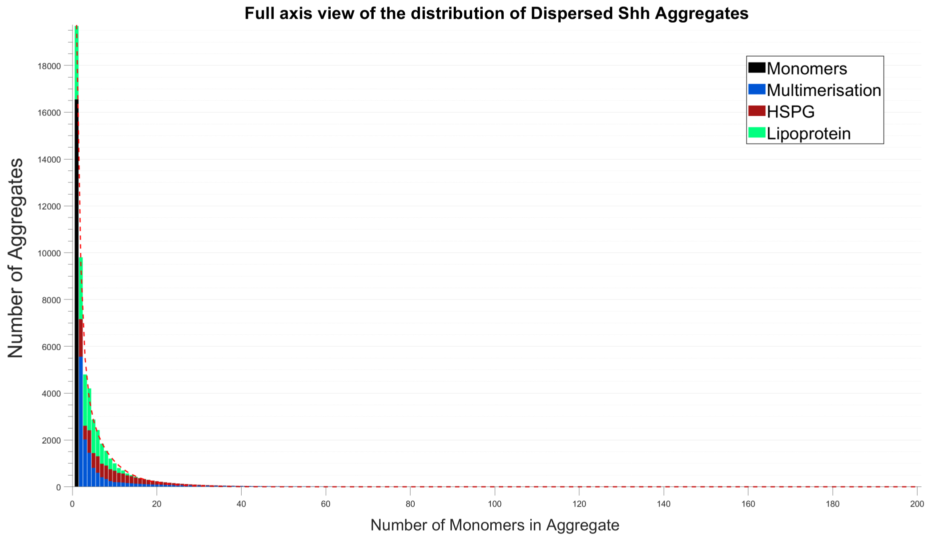

S5 Fig: Full axis view of the Dispersed Shh distribution. Figures show the full distribution of dispersed Shh aggregates that consist of up to 200 monomers. Simulation shown is at 24 hours.
