# Supplementary material for: A mathematical model of the role of aggregation in sonic hedgehog signalling
Source: PLoS Comput Biol. 2021 Feb 22;17(2):e1008562. doi: 10.1371/journal.pcbi.1008562 (PMC7932509; doi:10.1371/journal.pcbi.1008562)
Supplement: S7 Text — (PDF) [file pcbi.1008562.s019.pdf]

A Mathematical Approach to Understanding the Role of Aggregation in Sonic Hedgehog Signalling

Supplementary Information

Daniel J. A. Derrick, Kathryn Wolton, Richard Currie and Marcus John Tindall

S7 Sensitivity analysis

We conducted a sensitivity analysis to observe the effect that variation in certain parameters has on aggregate production. We found that increasing the source values for HSPGs or lipoproteins benefited the respective mechanisms by increasing the quantity of aggregates that could be produced by each process. This would consequently increase the competition within the mechanism; an increased number of aggregates recruit from the same population, depleting it at a greater rate and ultimately leads to a reduction in the average number of Shh that associates into the respective aggregates. In both scenarios multimer formation would be impeded. This is because an increase in lipoproteins would subsequently broaden their competition for Shh monomers and an increased quantity of HSPG aggregates would promote a greater rate of multimer recruitment. However, whereas an increase in the HSPG source rate would benefit lipoproteins by reducing multimer formation, an increase in lipoproteins would hinder HSPG aggregation, which is a result of the decreased rates of multimerisation.

Changes to the rate of dispersal was summarised in the main text and the distribution produced following the complete removal of dispersal was given in Fig 6. Increasing the rate of dispersal severely disrupted the overall production of aggregates, and impacted multimerisation least. This is most likely due to the increased removal of free particles by which HSPGs and lipoproteins are required to utilise in the formation of aggregates. Multimerisation is distinct from this and instead is not reliant on these particles. A reduction in rate of dispersal leads to an opposite effect. Aggregate production by the lipoprotein mechanism was dominant when compared to the remaining mechanisms. Because of this, multimer production was significantly inhibited due to the greater competition for Shh monomers by lipoprotein recruitment. The increased retaining of HSPGs at the cell surface as a result of a reduced sink is further detrimental to multimer formation. This is because the increased number of HSPGs cause a greater rate of multimer recruitment.

However, as multimers are formed at reduced rates and with a lesser monomer availability as a result of the aforementioned outcomes, the HSPG mechanism has an overall minimal benefit when dispersal rates are increased.

Increasing the rate of multimerisation enhances multimer production and consequently HSPG aggregate formation. The lipoprotein mechanism is subject to a greater degree of competition for monomers from multimerisation and thus aggregate production with lipoproteins is impeded. An increased rate of HSPG production benefits the respective mechanism in forming greater sizes but reduces multimer production due to increased recruitment. This however enhances the formation of aggregates by lipoproteins, which profits from reduced competition by multimers. Lastly, increasing the rate of lipoprotein aggregate formation disrupts both the HSPG and multimerisation mechanism through increased competition for monomers and reducing multimer production.

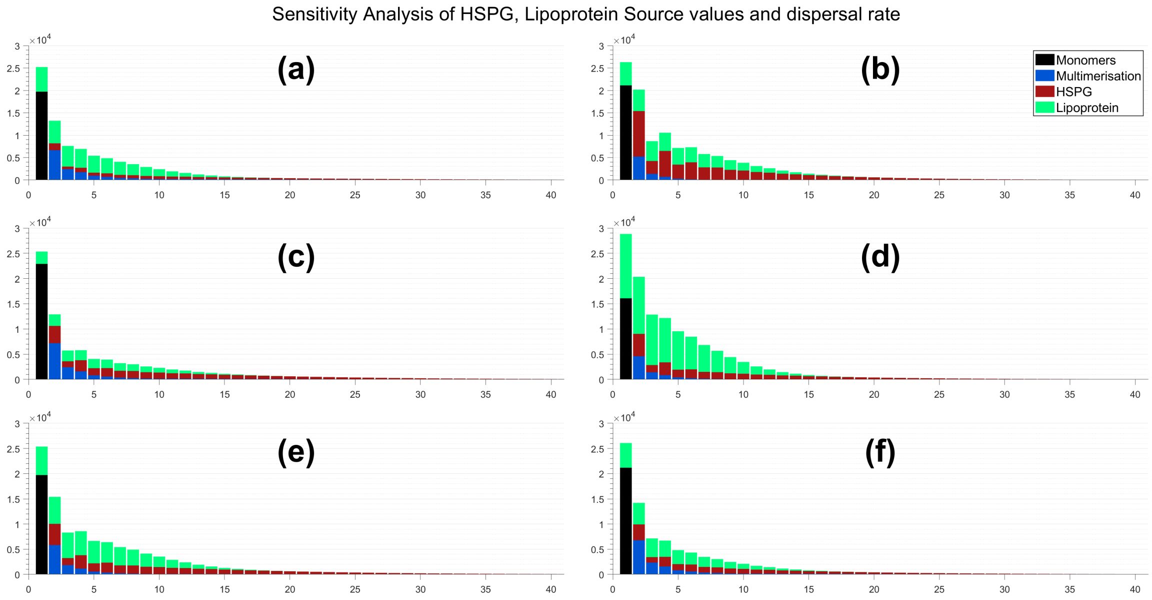

S7 Fig: Shh aggregate distributions with: (a) half the source of HSPGs; (b) doubled source of HSPGs; (c) half the source of lipoproteins; (d) doubled source of lipoproteins; (e) half rate of dispersal; and (f) doubled rate of dispersal.

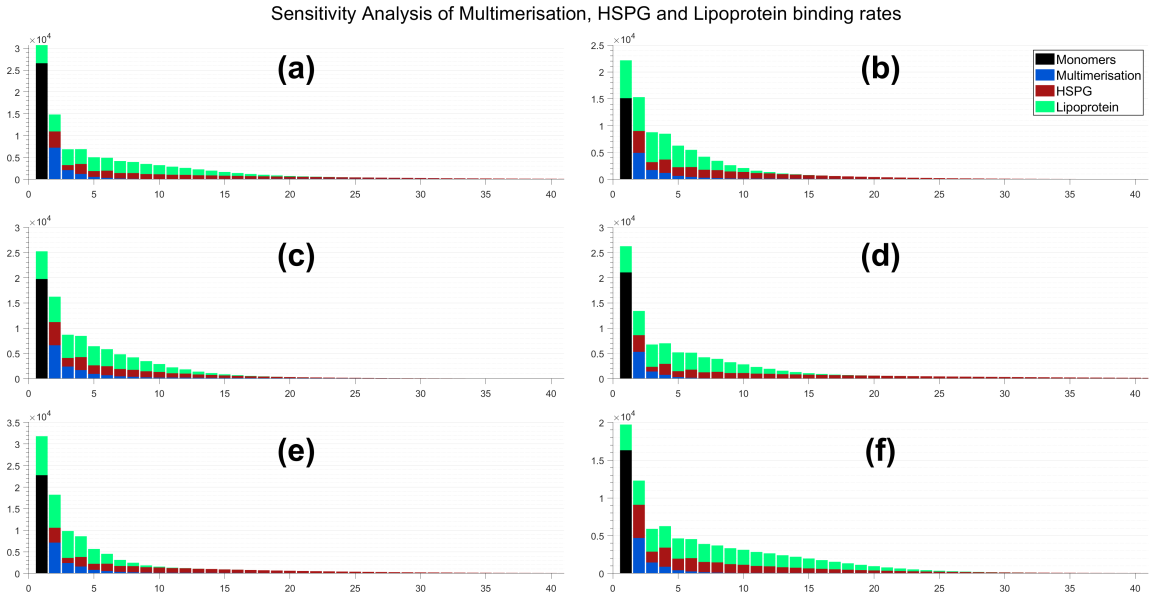

S8 Fig: Shh aggregate distributions with: (a) half the rate of multimerisation; (b) doubled rate of multimerisation; (c) half the rate of HSPG binding; (d) doubled rate of HSPG binding; (e) half the rate of lipoprotein binding; and (f) double the rate of lipoprotein binding.
