# Supplementary material for: A mathematical model of the role of aggregation in sonic hedgehog signalling
Source: PLoS Comput Biol. 2021 Feb 22;17(2):e1008562. doi: 10.1371/journal.pcbi.1008562 (PMC7932509; doi:10.1371/journal.pcbi.1008562)
Supplement: S8 Text — (PDF) [file pcbi.1008562.s020.pdf]

A Mathematical Approach to Understanding the Role of Aggregation in Sonic Hedgehog Signalling

Supplementary Information

Daniel J. A. Derrick, Kathryn Wolton, Richard Currie and Marcus John Tindall

S8 Diffusion coefficient approximation

Produced aggregates are likely to have considerably differing rates of diffusion between the mechanisms. In addition, the continued binding of Shh will impact aggregates, and the degree of this will vary dependent on the mechanism by which the aggregate is produced. In the following we seek to explore this using estimates and available literature sources.

To calculate diffusion coefficient for Shh aggregates produced via multimerisation, HSPG and lipoprotein recruitment, we make assumptions for the structure by which each are made. In the follow discussions we utilise a diameter for Shh monomer of 4 nm, which is as noted by Koleva and colleagues [1].

Multimerisation

To calculate estimations for Shh multimer diffusion coefficients we first consider approximations for a Shh monomer, a multimer consisting of 6 Shh proteins (hexamer) and a multimer that consists of 36 monomers (36-mer). We aim to model each as a sphere and calculate the diffusion coefficient using the Stokes-Einstein equation given by Equation 1, for which we are required to estimate the radius of each. For monomers we assumed that a Shh protein can be modelled as a sphere which has a radius of 2 nm. We next assume that hexamers can be considered as comprising of equally arranged monomers forming as sphere, as depicted in S9(a) Fig. This structure would therefore have a radius that is equal to the diameter of a monomer, which is 4 nm. For a 36-mer we continue this approach and assume that the larger aggregate can be viewed as a composition of Shh hexamers that are arranged equally around a center, as is shown in S9(b) Fig. This multimer is therefore assumed to have the radius of two Shh monomer diameters (8 nm).

We calculated the diffusion coefficient of a monomer, hexamer and 36-mer by using the Stokes-Einstein equation, which approximates the diffusion of spherical particles through a liquid medium at a constant temperature. The Stokes-Einstein equation is given by,

$$D = \frac{kT}{6\pi\eta r}, \tag{1}$$

where k is the Boltzmann's constant, T is the temperature in Kelvin, η is the viscosity of the liquid medium and r is the radius of the spherical particle. For our calculations we assume that the fluid through which Shh aggregates diffuse has a viscosity equivalent to water, which, at a temperature of 298.15 Kelvin (25° Celsius), is 8.9×10^{-4} Pa·.

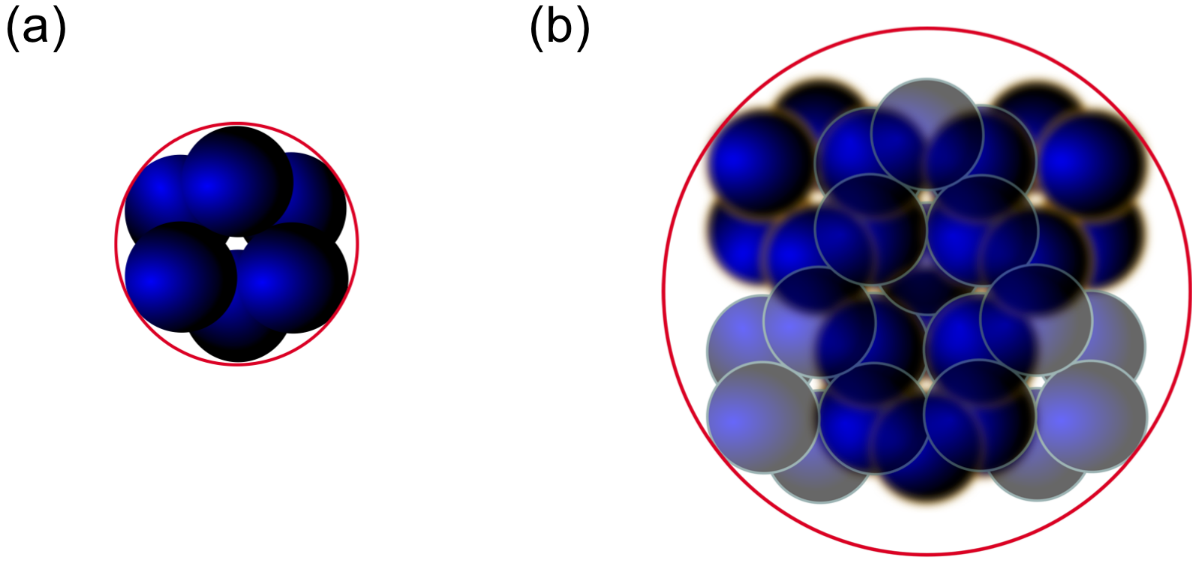

S9 Fig: Diagram of Shh multimers: We assume a hexamer **(a)** can be modelled as a sphere with the radius of a Shh monomer and a 36-mer **(b)** can be modelled as a sphere that has a radius equal to the diameter of a hexamer.

Using the curve fitting toolbox (cftool) in MATLAB [2] we find the coefficients for a quadratic equation that is sufficient to fit the diffusion coefficients calculated for the monomer, hexamer and 36-mer. This allows us to estimate the coefficients for remaining multimers.

Lipoproteins

The particular class of lipoproteins that is likely to bind Shh for transport has not been identified. For our calculations we considered the binding of low-density lipoproteins

(LDL) as this subspecies is sufficiently large to be occupied by the amounts of Shh binding that we consider and still be comparable in overall size to the opposing mechanisms.

To calculate the diffusion coefficient of lipoprotein aggregates we make the assumption that the binding of Shh to lipoproteins creates a ‘layer’ of Shh monomers on the surface. Large amounts of Shh binding to lipoproteins creates a solid layer around the particle which creates an overall larger sphere with a greater radius. Shh binding to lipoproteins is depicted in S10 Fig.

From Tindall et al. [3] we have that the radius of an LDL particle is 10 nm and therefore an LDL bound by a maximum amount of Shh has a radius of 14 nm (radius added to the diameter of a Shh monomer).

Using the Stokes-Einstein equation (Equation (5)) we are able to calculate an approximate diffusion coefficient for both a lipoprotein that is not bound by Shh and one that is bound with a maximum amount of Shh. By assuming that the latter estimate corresponds to the binding of 40 Shh monomers, we utilise a data fitting via MATLAB [2] to estimate the diffusion coefficients for remaining lipoprotein aggregate sizes.

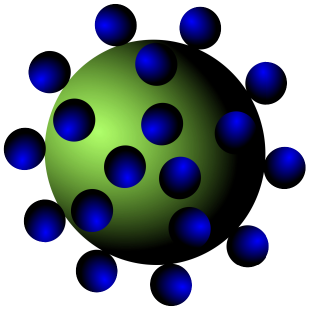

S10 Fig: Diagram of Shh aggregation by association with lipoproteins: Here we assume that Shh monomers bind a lipoprotein individually and contribute to form a ‘layer’ upon the particle.

HSPGs

Creating an assumption for the shape of Shh-HSPG aggregates is especially difficult due to the ambiguous role for which it promotes Shh aggregate formation. The assumption we elect to make follows in part from the chain-like structures that were considered by Whalen and colleagues [4]. As we depict in S11 Fig, HSPG aggregates are viewed as ‘tubular’ chain-like structures. We follow a similar approach as with the mechanisms described previously and assume that Shh bound HSPGs are in the form of various hexamers which bind opposing sides of a HSPG chain. In this sense a HSPG aggregate that is bound by 36 Shh proteins is to be modelled as a cylinder consisting of 6 hexamers divided across

opposing sides of the heparin chain. The radius of this cylinder will be be equal to the diameter of a hexamer which we discussed and calculated above. The length of cylinder can also be found to be equal to the diameter of 3 hexamers.

This process was applied to HSPG aggregates with 12, 24 and 36 Shh monomers associated, which meant cylinder lengths of 8nm, 16nm and 24nm respectively, to calculate an approximate volume. We also considered a HSPG aggregate with a sole hexamer bound and assumed the volume would be equal to that if it were not bound to a HSPG. With these volumes we calculated approximations to the diffusion coefficient using Equation (1), and applied Matlab's curve fitting toolbox to estimate the values for the remaining HSPG aggregate sizes. We arrive at the estimates for the diffusion coefficients for the aggregates produced by different mechanisms and composition of monomers as shown in S12 Fig.

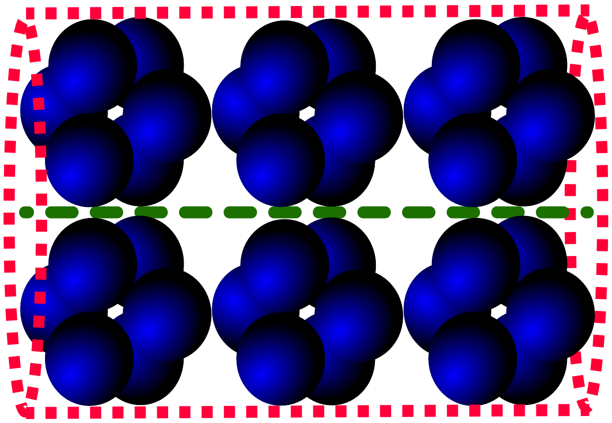

S11 Fig: Diagram of Shh aggregation by association with HSPGs: We assume that Shh multimers bind HSPGs as hexamers to form a structure that can be modelled as a cylinder.

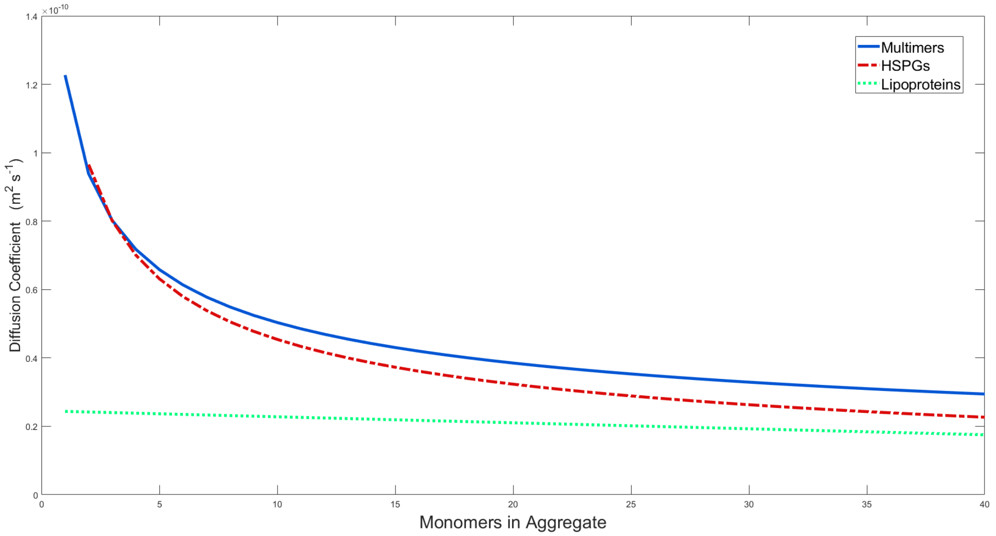

S12 Fig: Approximated diffusion coefficients for the aggregates produced by multimerisation, HSPG and lipoprotein recruitment.

References

1. Koleva MV, Rothery S, Spitaler M, Neil MAA, Magee AI. Sonic hedgehog multimerization: A self-organizing event driven by post-translational modifications? Molecular Membrane Biology. 2015;32(3):6574. doi:10.3109/09687688.2015.1066895.

2. Matlab. The MathWorks; Release 2018b. Natick, Massachusetts, United States.

3. Tindall MJ, Wattis JAD, OMalley B, Pickersgill L, Jackson KG. A continuum receptor model of hepatic lipoprotein metabolism. Journal of Theoretical Biology. 2009;257(3):371384. doi:10.1016/j.jtbi.2008.11.016.

4. Whalen DM, Malinauskas T, Gilbert RJC, Siebold C. Structural insights into proteoglycan-shaped Hedgehog signaling. PNAS. 2013;110(41):1642016425. doi:10.1073/pnas.1310097110.
