# Supplementary material for: A mathematical model of the role of aggregation in sonic hedgehog signalling
Source: PLoS Comput Biol. 2021 Feb 22;17(2):e1008562. doi: 10.1371/journal.pcbi.1008562 (PMC7932509; doi:10.1371/journal.pcbi.1008562)
Supplement: S1 Table — (PDF) [file pcbi.1008562.s021.pdf]

A Mathematical Approach to Understanding the Role of Aggregation in Sonic Hedgehog Signalling

Supplementary Information

Daniel J. A. Derrick, Kathryn Wolton, Richard Currie and Marcus John Tindall

Parameter	Definition	Value	Source
α	Source of monomers	1.152×10^6 monomers/day	[?]
δ	Source of HSPGs	4.60×10^4 particles/day	Estimate.
γ	Source of lipoproteins	6.00×10^4 particles/day	Estimate.
β	Rate of dispersal	0.75 day^{-1}	Estimate.
$m_{a,b}$	Multimerisation binding rate	6.00×10^{-4} (multimer day)$^{-1}$	Estimate.
h_i	Multimer to HSPG binding rate	4.00×10^{-4} (multimer day)$^{-1}$	Estimate.
k_i	Monomer to lipoprotein binding rate	5.00×10^{-4} (protein day)$^{-1}$	Estimate.

S1 Table: Parameters for the aggregation model.
